# OTUD6B-AS1 Might Be a Novel Regulator of Apoptosis in Systemic Sclerosis

**DOI:** 10.3389/fimmu.2019.01100

**Published:** 2019-05-17

**Authors:** Miki Takata, Elena Pachera, Mojca Frank-Bertoncelj, Anastasiia Kozlova, Astrid Jüngel, Michael L. Whitfield, Shervin Assassi, Maurizio Calcagni, Jeska de Vries-Bouwstra, Tom W. Huizinga, Fina Kurreeman, Gabriela Kania, Oliver Distler

**Affiliations:** ^1^Department of Rheumatology, Center of Experimental Rheumatology, University Hospital Zürich, Zurich, Switzerland; ^2^Department of Molecular and Systems Biology, Geisel School of Medicine at Dartmouth, Hanover, NH, United States; ^3^Department of Internal Medicine, Division of Rheumatology, The University of Texas Health Science Center at Houston, McGovern Medical School, Houston, TX, United States; ^4^Department of Plastic Surgery and Hand Surgery, University Hospital Zürich, Zurich, Switzerland; ^5^Department of Rheumatology, Leiden University Medical Center, Leiden, Netherlands

**Keywords:** systemic sclerosis, ovarian tumor domain containing 6B-antisense RNA1 (OTUD6B-AS1), antisense long non-coding RNA (AS lncRNA), cyclin D1, proliferation, apoptosis, dermal fibroblasts, human pulmonary artery smooth muscle cells

## Abstract

Antisense long non-coding RNAs (AS lncRNAs) have increasingly been recognized as important regulators of gene expression and they have been found to play key roles in several diseases. However, very little is known about the role of AS lncRNAs in fibrotic diseases such as systemic sclerosis (SSc). Our recent screening experiments by RNA sequencing showed that ovarian tumor domain containing 6B antisense RNA1 (OTUD6B-AS1) and its sense gene OTUD6B were significantly downregulated in SSc skin biopsies. Therefore, we aimed to identify key regulators of OTUD6B-AS1 and to analyze the functional relevance of OTUD6B-AS1 in SSc. OTUD6B-AS1 and OTUD6B expression in SSc and healthy control (HC) dermal fibroblasts (Fb) after stimulation with transforming growth factor-β (TGFβ), Interleukin (IL)-4, IL-13, and platelet-derived growth factor (PDGF) was analyzed by qPCR. To identify the functional role of OTUD6B-AS1, dermal Fb or human pulmonary artery smooth muscle cells (HPASMC) were transfected with a locked nucleic acid antisense oligonucleotide (ASO) targeting OTUD6B-AS1. Proliferation was measured by BrdU and real-time proliferation assay. Apoptosis was measured by Caspase 3/7 assay and Western blot for cleaved caspase 3. While no difference was recorded at the basal level between HC and SSc dermal Fb, the expression of OTUD6B-AS1 and OTUD6B was significantly downregulated in both SSc and HC dermal Fb after PDGF stimulation in a time-dependent manner. Only mild and inconsistent effects were observed with TGFβ, IL-4, and IL-13. OTUD6B-AS1 knockdown in Fb and HPASMC did not affect extracellular matrix or pro-fibrotic/proinflammatory cytokine production. However, OTUD6B-AS1 knockdown significantly increased Cyclin D1 expression at the mRNA and protein level. Moreover, silencing of OTUD6B-AS1 significantly reduced proliferation and suppressed apoptosis in both dermal Fb and HPASMC. OTUD6B-AS1 knockdown did not affect OTUD6B expression at the mRNA level and protein level. Our data suggest that OTUD6B-AS1 regulates proliferation and apoptosis via cyclin D1 expression in a sense gene independent manner. This is the first report investigating the function of OTUD6B-AS1. Our data shed light on a novel apoptosis resistance mechanism in Fb and vascular smooth muscle cells that might be relevant for pathogenesis of SSc.

## Introduction

Systemic sclerosis (SSc) is an autoimmune disease characterized by immune abnormalities, microvascular dysfunction, and fibrosis in the skin and multiple internal organs. Patients are sub-classified into limited cutaneous systemic sclerosis (lcSSc) and diffuse cutaneous systemic sclerosis (dcSSc), based on the extent of skin involvement. Fibrosis in multiple internal organs is an important contributor to the high mortality in SSc. Upregulation of profibrotic cytokines such as PDGF and TGFβ and their respective signaling pathways is a key feature of SSc (1, 2). Microvascular manifestations include loss of capillaries and vessel wall thickening of small arteries leading to reduced perfusion and hypoxia. Major clinical manifestations of the microvasculopathy are digital ulcers and pulmonary arterial hypertension (PAH). PAH is another major contributor to death in SSc patients. However, the molecular pathogenesis of SSc leading to these manifestations is still not fully understood and there is no registered approved anti-fibrotic therapy available to date (3–5).

Long non-coding RNAs (lncRNAs) represent a class of transcripts longer than 200 nucleotides that are not translated into proteins. LncRNAs have been classified by their genomic location into intergenic lncRNA, intronic lncRNA, bidirectional lncRNA, enhancer lncRNA, sense lncRNA, and antisense (AS) lncRNA (6). AS lncRNAs are transcribed from the opposite strand of protein-coding genes and overlap one or several exons and introns with the sense gene. High-throughput RNA sequencing analysis showed that for most AS lncRNAs the expression is about 10-fold lower than for their coding gene (7, 8). Moreover, the expression of AS lncRNAs is more tissue specific than those of protein coding genes (8–10). In recent years, AS lncRNAs have increasingly been recognized as important regulators of their sense gene expression. However, some AS lncRNAs can exert their biologic effects independently of the sense gene. For example, NKX2-1-AS1 is an AS lncRNA that has been linked to human lung carcinoma. NKX2-1-AS1 regulates CD274, the gene encoding the Programmed Death-Ligand 1 (PD-L1), and cell-cell interaction genes, but not the adjacent protein-coding gene NKX2-1 (11). In general, AS lncRNAs can function either in *cis* or in *trans*. In *cis*, lncRNAs regulate the expression of transcription sites on the same chromosome. In *trans*, lncRNAs regulate the gene expression on other chromosomes (9, 12). AS lncRNAs affect almost all stages of gene expression processes via pre-transcriptional, co-transcriptional, or post-transcriptional mechanisms (12–14).

Recently, a variety of studies have reported that AS lncRNAs play key roles in the pathogenesis of different diseases including cancers (15–17), cardiac vascular diseases (6, 18), kidney diseases (18), and central nervous system diseases (19). However, only very few AS lncRNAs have been linked to SSc and fibrosis (20, 21). Previous RNA sequencing analysis of SSc and healthy control (HC) skin biopsies performed by our groups revealed 676 differentially expressed non-coding genes, 38% of these non-coding genes were classified as antisense genes. The differentially expressed antisense genes included ovarian tumor domain containing 6B (OTUD6B)-AS1 (22).

The sense gene, OTUD6B, encodes for a deubiquitinating enzyme. Deubiquitinating enzymes are classified into five families based on the architecture of their catalytic domains. OTUD6B belongs to the ovarian tumor proteases (OTUs) family. Additional families are ubiquitin specific proteases (USPs), ubiquitin COOH-terminal hydrolases (UCHs), Josephins, and the JAB1/MPN/MOV34 family (JAMMs) (23). It has been shown that deubiquitinating enzymes have crucial roles in many biological processes, including cell cycle regulation, apoptosis, and DNA repair. In particular, they can regulate specific molecular pathways such as Wnt/β-catenin and NFκB signaling (24).

**Figure 1:**
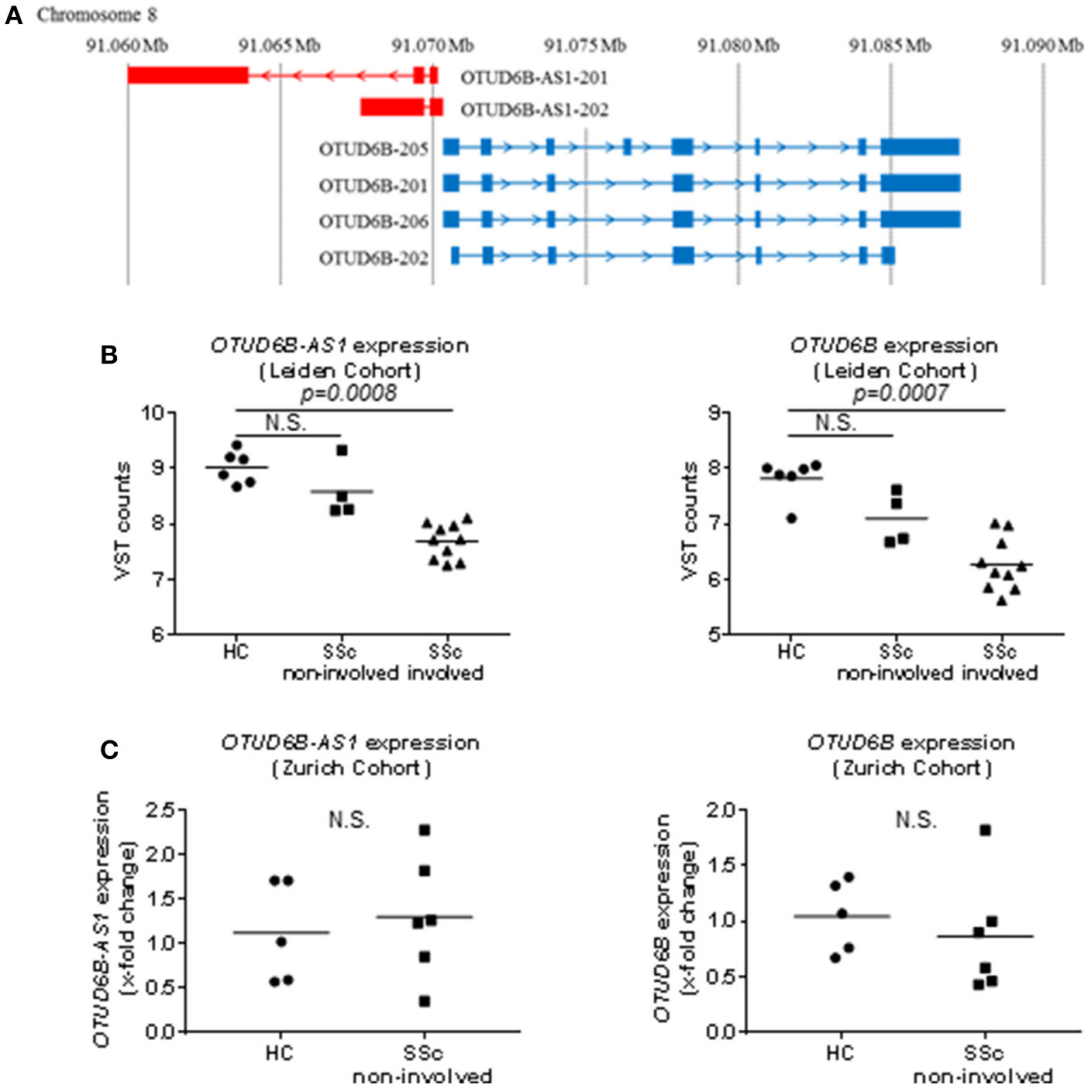
Genomic localization and basal expression in skin of OTUD6B-AS1 and OTUD6B. **(A)** OTUD6B-AS1 and OTUD6B are located on chromosome 8 and oriented head-to-head (GENCODE v27). **(B)** In the Leiden cohort, OTUD6B-AS1, and OTUD6B expression was analyzed in healthy control (HC), non-involved and involved skin of systemic sclerosis patients (SSc) using next generation sequencing [HC: *n* = 6, SSc (non-involved): *n* = 4, SSc (involved): *n* = 10, (22)]. **(C)** In the Zurich cohort, OTUD6B-AS1, and OTUD6B expression was measured in HC and non-involved SSc patients skin by qPCR (HC: *n* = 5, SSc (non-involved): *n* = 6). Expression level was normalized using *RPLP0*. **(C)**. Data are shown as single values and mean. Statistical analysis was performed by unpaired *t*-test. N.S, not significant.

Little is known about the function of OTUD6B. OTUD6B has been suggested to be involved in cell cycle regulation in B lymphocytes after prolonged cytokine stimulation and in DNA synthesis in non-small cell lung cancer cells (25, 26). Moreover, defective OTUD6B function has been associated with cognitive dysfunction and dysmorphic features in development (27). There is no information available about the function of the antisense lncRNA, OTUD6B-AS1. OTUD6B-AS1 is transcribed from the opposite strand of the OTUD6B gene, which is located on chromosome 8 in head-to-head orientation to OTUD6B-AS1 (Figure 1A).

Here, we analyzed the differential expression of OTUD6B-AS1 in SSc skin biopsies, identified pro-inflammatory and pro-fibrotic regulators of OTUD6B-AS1 expression, and defined the functional roles of OTUD6B-AS1 in the pathophysiology of SSc. In particular, we focused on OTUD6B-AS1 role in proliferation and apoptosis of human dermal fibroblasts and human pulmonary artery smooth muscle cells (HPASMC), which represent the two major cellular players in fibrosis and vasculopathy of SSc.

## Methods

### Cell Culture

Skin biopsies were obtained from the forearm of SSc patients at the Department of Rheumatology, University Hospital Zurich, Switzerland or at the Department of Internal Medicine, Division of Rheumatology, University of Texas Health Science Center, Houston, USA. All patients fulfilled the American College of Rheumatology (ACR)/European League Against Rheumatism (EULAR) 2013 criteria for SSc. Healthy donors were obtained from the Department of Plastic Surgery and Hand Surgery of the University Hospital Zurich. Donors characteristics are listed in Table 1. Patients and healthy subjects from the Leiden and Dartmouth cohort have been described elsewhere (22). In general, skin areas with clinically detectable fibrotic changes are referred to as “involved skin,” and skin areas without clinically detectable fibrotic changes are referred to as “non-involved skin.” This study was carried out in accordance with the recommendations of World Medical Association Declaration of Helsinki, ICH-GCP guidelines or ISO 14155. The study was approved by the Ethics Committee of the Canton of Zurich (approved ethical applications KEK-ZH 515, PB-2016-02014, and KEK-Nr. 2018-01873). All subjects gave written informed consent in accordance with the Declaration of Helsinki.

**Table 1:** Characteristics of systemic sclerosis (SSc) patients and healthy control (HC) donors in the Zurich cohort.

**Donor**	**Gender**	**Site of biopsy**	**Disease subtype**	**Disease duration (years)**	**mRSS**	**Fibrosis at the site of biopsy**	**Skin biopsy expression analysis**	**PDGF stimulation/ functional assays**
SSc 1	Female	Forearm	Diffuse	10.16	4	No	x	x
SSc 2	Male	Forearm	Diffuse	1.32	16	Yes	x	x
SSc 3	Female	Forearm	Diffuse	9.16	6	No	x	
SSc 4	Male	Forearm	Diffuse	0.72	n.a.	n.a.		x
SSc 5	Male	Forearm	Limited	n.a.	0	No	x	
SSc 6	Female	Forearm	Limited	6.0	0	No	x	
SSc 7	Female	Forearm	Limited	n.a.	0	No	x	x
SSc 8	Female	Forearm	Limited	n.a.	6	No		x
SSc 9	Female	Forearm	Limited	n.a.	3	No		x
HC 1	Female	Abdomen					x	
HC 2	Female	Breast					x	
HC 3	Female	Hernia surgery					x	
HC 4	Male	Forearm						x
HC 5	Male	Forearm					x	
HC 6	Male	Forearm					x	x
HC 7	Male	Forearm					x	x
HC 8	Female	Breast						x
HC 9	Male	Forearm						x
HC 10	Male	Forearm						x
HC 11	Female	Forearm						x
HC 12	Female	Forearm						x
HC 13	Female	Forearm						x
HC 14	Male	Forearm						x

*Skin biopsies were obtained from patients fulfilling the American College of Rheumatology (ACR)/European League against Rheumatism (EULAR) 2013 criteria. The disease subset was determined according to the criteria proposed by LeRoy et al. (28). Disease duration was measured from the onset of the first non-Raynaud symptoms attributable to SSc. n.a, not available*.

Human dermal fibroblasts (Fb) were isolated from SSc and site-matched HC skin biopsies by outgrowth culture. Dermal Fb were maintained in Dulbecco's modified eagle's medium (DMEM, Sigma) supplemented with 10% fetal bovine serum (FBS), 50 units/ml of penicillin, 50 μg/ml of streptomycin and 2 μl/ml 2-mercaptoethanol. Fb from passages 4 to 11 were used for all experiments.

Human pulmonary artery smooth muscle cells (HPASMC) were purchased from ScienCell and maintained in smooth muscle cell medium (SMCM, ScienCell) supplemented with 1% FBS, 100 units/ml of penicillin, 100 μg/ml of streptomycin, and smooth muscle cell growth supplement (all, ScienCell). HPASMC from passages 4 to 9 were used in all experiments.

Dermal Fb and HPASMC were cultured at 37°C in a humidified 5% CO_2_-containing atmosphere. Dermal Fb and HPASMC were stimulated with transforming growth factor-β (TGFβ; 10 ng/ml, PeproTech), Interleukin (IL)-4 (10 ng/ml, ImmunoTools), IL-13 (10 ng/ml, ImmunoTools), and platelet-derived growth factor (PDGF; 20 ng/ml, PeproTech) for 6, 24, 48, and 72 h.

### Cell Fractionation

To detect the localization of lncRNA OTUD6B-AS1, dermal Fb were fractionated as previously described (29). Cells were trypsinized, lysed with hypotonic lysis buffer (10 mM Tris-HCl pH 7.5, 10 mM NaCl, 3 mM MgCl_2_, 0.3% NP-40 and 10% glycerol) supplemented with RNase inhibitor (SUPERase-In, Thermo Fisher Scientific) and incubated on ice for 20 min. Cells were then centrifuged at 10,00 g at 4°C for 3 min. Supernatant (cytoplasmic fraction) was taken and RNA precipitated in 150 mM ethanol sodium acetate at −20°C for 1 h. Cell pellets (nuclear fraction) were washed with hypotonic lysis buffer and centrifuged at 200 g at 4°C for 2 min. Precipitated cytoplasmic RNA was pelleted, washed in ice-cold 70% ethanol and centrifuged at 17,000 g at 4°C for 5 min. Finally, Trizol was added to both fractions and RNA was extracted.

### Transient Transfection of Dermal Fb and HPASMC

To identify the function of the lncRNA OTUD6B-AS1, HC Fb, or HPASMC were transfected with locked nucleic acid antisense oligonucleotide (ASO, QIAGEN) targeting OTUD6B-AS1 (k.d.: 5′- GAATGAAATAGACGTT−3′, k.d._2: 5′-TTCAGTAATTCGATT-3′, k.d._3: 5′-GGACCAAAATCAAAGA-3′, final concentration of 25 nM for dermal Fb and 50 nM for HPASMC) using a concentration of 2 μl/ml Lipofectamine 2,000 transfection reagent according to the manufacturer's protocol (Thermo Fisher Scientific). Cells were harvested for gene and protein expression analysis 24, 48, 72 h after transfection or prepared for functional assays.

### RNA Isolation and cDNA Synthesis

Total RNA was extracted from cultured cells using the High Pure RNA Isolation Kit (Roche) according to the manufacturer's instructions. RNA concentration of isolated RNA was assessed using spectrophotometer (NanoDrop). For complementary DNA (cDNA) synthesis, 200 ng of total RNA was reverse-transcribed using random hexamers and reverse transcriptase using the Transcriptor First Strand cDNA Synthesis Kit (Roche).

### RNA Sequencing and Quantitative Real-Time PCR (qPCR) Analysis

RNA sequencing performed on the skin biopsies from Leiden has been described recently (22). Total RNA extracted from skin biopsies from Zurich as well as functional *in vitro* experiments were analyzed by qPCR (Stratagene Mx3005P QPCR system, Agilent Technologies) using SYBR Green Master Mix (Promega). The expression of glyceraldehyde-3-phosphate dehydrogenase (GAPDH) and ribosomal protein lateral stalk subunit P0 (RPLP0) was used to normalize the obtained expression levels for the gene of interest. Primer sequences are listed in the (Supplementary Table 1). Specific amplification was verified measuring dissociation curves. Differential gene expression was calculated using the comparative threshold cycle method (ΔΔCt method) (30).

### Western Blotting

Cells were lysed using ice-cold RIPA buffer (Sigma) supplemented with phosphatase inhibitor cocktail (PhosSTOP, Roche) and protease inhibitor cocktail (cOmplete ULTRA Tablets, Roche). Insoluble material was removed by centrifugation at 12,000 rpm, 20 min at 4°C. Whole cell lysates were separated on 10 or 12% SDS polyacrylamide gel electrophoresis (SDS-PAGE) and proteins were transferred by electroblotting onto nitrocellulose membranes (Amersham Protran, GE Healthcare). Membranes were blocked with 5% non-fat milk in Tris-buffered saline containing 0.1% Tween20 (TBS-T) and incubated for 1 h. Western blots were performed using rabbit anti OTUD6B antibody (Abcam, #ab127714, 1:3,000), rabbit anti Cyclin D1 antibody (Cell signaling, #2978, 1:1,000), rabbit anti Cyclin D2 antibody (Cell signaling, #3741, 1:1,000), rabbit anti c-MYC antibody (Cell signaling, #13987, 1:1,000), rabbit anti Caspase 3 antibody (Cell signaling, #9662, 1:1,000) which detects both cleaved and uncleaved forms of caspase 3, rabbit anti E2F1 antibody (Abcam, #ab179445, 1:1,000), and rabbit anti GAPDH antibody (Cell signaling, #2118, 1:10,000). Bands were detected using goat anti rabbit secondary antibodies conjugated to horseradish peroxidase (Abcam, #ab6721, 1:5000). Cyclin D1, Cyclin D2, c-MYC, E2F1 expression was normalized to the expression of GAPDH, and cleaved caspase 3 expression was normalized to uncleaved caspase 3 expression. Calculation of the relative expression of protein was performed using ImageJ software.

### BrdU Cell Proliferation ELISA

To quantify cell proliferation, dermal Fb, and HPASMC were seeded in 96-well plates at a density of 2,000 cells per well and transfected with the ASO negative control or ASO targeting OTUD6B-AS1. Seventy-two hours after transfection, 5-bromo-2′-deoxyuridine (BrdU) was added to each well and incubated for four additional hours. Incorporated BrdU was detected using the BrdU (colorimetric) cell proliferation ELISA kit (Roche/Sigma-Aldrich) according to the manufacturer's instructions.

### Real-Time Monitoring of Proliferation

Real-time monitoring of cell proliferation was performed using the xCelligence RTCA DP system (ACEA Biosciences). Background impedance was measured 30 min after adding 100 μl of culture medium to E-plates 16 PET (ACEA Biosciences). Next, either 2,500 dermal Fb or 3,000 HPASMC per 100 μl of culture medium per well were seeded. After 30 min incubation at room temperature, plates were placed into the xCelligence system. The relative change in electrical impedance termed the Cell Index (CI) was measured. Fb and HPASMC were transfected with negative control or ASO targeting OTUD6B-AS1 26 h or 50 h after seeding, respectively. CI was monitored every 5 min from 0 h to 13 h, every 15 min from 13 h to 25 h and every 30 min from 25 h until CI reached plateau. CI was normalized at the time of transfection for every experiment. The RTCA software 2.0 (ACEA Biosciences) was used to calculate the slope of the CI curve as measure of cell proliferation.

### Caspase 3/7 Assay

To measure caspase-3 and caspase-7 activities, dermal Fb, and HPASMC were seeded in 96-well plates at a density of 2,000 cells per well and transfected with either ASO negative control or ASO targeting OTUD6B-AS1 (final concentration of 25 nM for dermal Fb and 50 nM for HPASMC) as described above. To induce caspase 3/7 activation, cells were treated with 1 μM staurosporin (STP) 16 h before starting the assay (31, 32). Forty-eight or seventy-two hours after transfection, equal volumes of Caspase-Glo® 3/7 Reagent (Promega) were added into the cell culture medium and incubated for additional 1.5 h at room temperature. Luminescence as a measure of caspase 3/7 activity was recorded by plate-reading luminometer (Synergy HT, BioTek).

### Statistics

All data are presented as mean ± standard deviation (SD). Paired samples were analyzed by two-tailed paired *t*-test, and unpaired samples were analyzed by unpaired *t*-test. Comparison of multiple groups was performed by one-way ANOVA with Dunnett's multiple comparisons test. *P* < 0.05 were considered statistically significant. All statistic tests were performed with GraphPad Prism version 7.0.

## Results

### OTUD6B-AS1 and OTUD6B Expression Is Downregulated in SSc Skin

RNA sequence analysis of 6 HC and 14 SSc patients skin biopsies performed at the Leiden University Medical Center showed that OTUD6B-AS1 expression was significantly downregulated in SSc skin and this was the most differentially expressed AS lncRNA. Moreover, the sense gene OTUD6B expression was also significantly downregulated in SSc skin (22). Interestingly, additional subanalysis showed that this downregulation in SSc skin was mostly seen in skin biopsies from involved skin, while non-involved skin did not show statistically significant changes as compared to healthy controls (Figure 1B). In addition, recently performed RNA sequencing analysis of additional 6 HC and 14 SSc biopsies performed at Geisel School of Medicine at Dartmouth, confirmed OTUD6B-AS1 as one of the top downregulated AS lncRNAs (22). The expression of OTUD6B-AS1 and OTUD6B was not changed in biopsies from the Zurich cohort, which only consisted of non-involved SSc skin biopsies as revealed by qPCR analysis (Figure 1C). Together, these data from different cohorts indicate that the expression of OTUD6B-AS1 and OTUD6B is downregulated in skin biopsies from SSc patients, mostly in clinically involved, fibrotic areas.

### OTUD6B-AS1 and OTUD6B Expression in Dermal Fb Is Tightly Regulated by PDGF

OTUD6B-AS1 and OTUD6B are strongly expressed in dermal Fb as well as in other cells types present in the skin (22). Therefore, we compared the expression levels of OTUD6B-AS1 and OTUD6B in dermal Fb. Under basal conditions, there was no statistically significant difference between HC and SSc Fb (Supplementary Figure 1).

Based on the expression in fibrotic areas in skin biopsies, we hypothesized that immunomodulatory cytokines involved in the pathophysiology of fibrosis might be important for the downregulation of OTUD6B-AS1 and OTUD6B. We therefore analyzed their time course response after stimulation with PDGF, TGFβ, IL-4 and IL-13.

**Figure 2:**
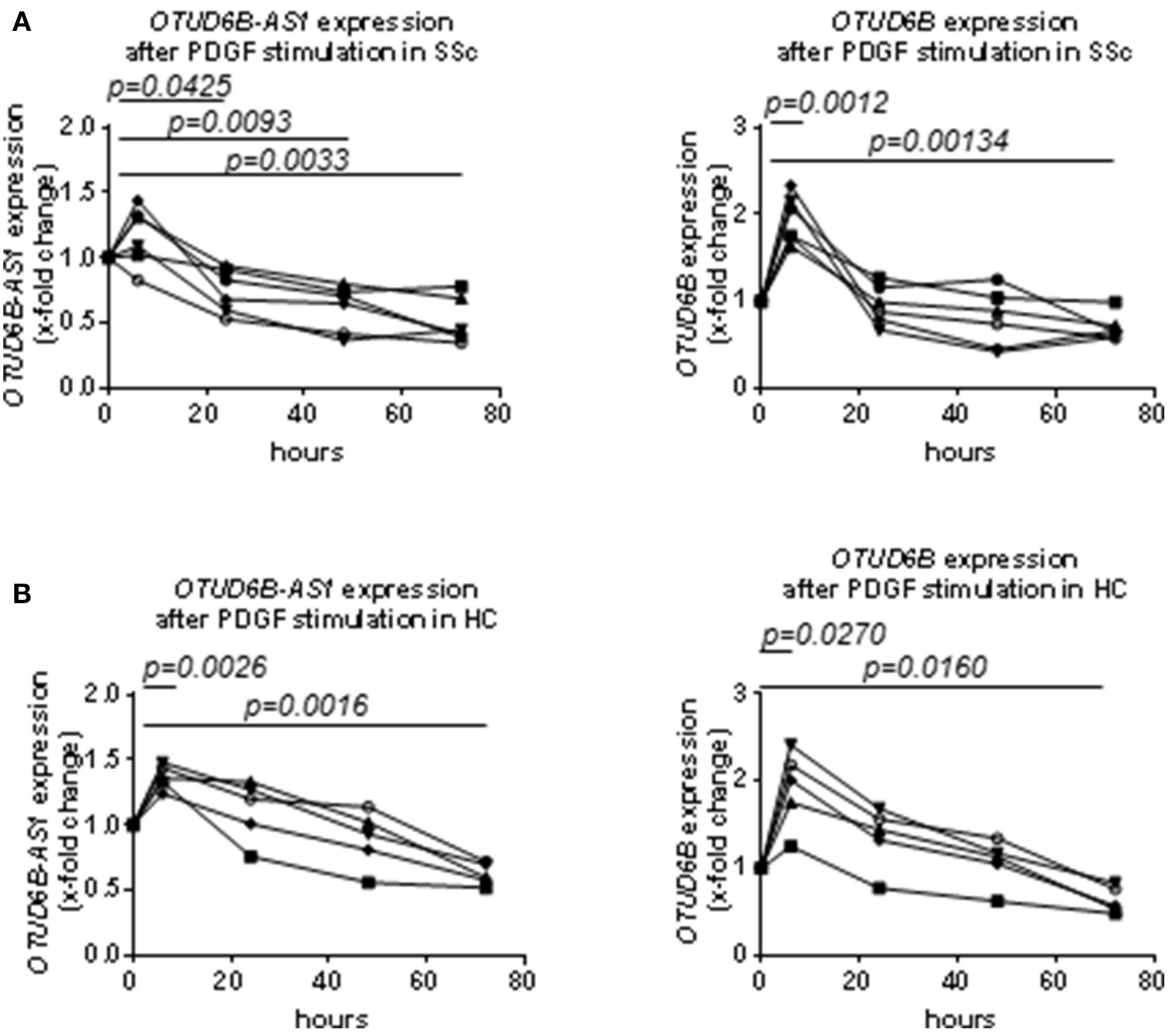
Time course analysis of the expression of OTUD6B-AS1 and OTUD6B in SSc and HC dermal Fb after platelet-derived growth factor (PDGF) stimulation. Dermal Fb were stimulated with PDGF (20 ng/ml) for 6, 24, 48, and 72 h. **(A)** Time course analysis of the expression of OTUD6B-AS1 and OTUD6B in SSc dermal Fb after platelet-derived growth factor (PDGF) stimulation (*n* = 6). **(B)** Time course analysis of the expression of OTUD6B-AS1 and OTUD6B in HC dermal Fb after platelet-derived growth factor (PDGF) stimulation (*n* = 5). Expression levels were measured by qPCR, normalized by GAPDH and RPLP0 and compared with non-stimulated dermal Fb. Data are shown as single values. Statistical analysis was performed by one-way ANOVA with Dunnett's multiple comparisons test.

OTUD6B-AS1 expression in dermal Fb from SSc patients was significantly downregulated after 24, 48, and 72 h of PDGF stimulation. OTUD6B expression was significantly upregulated after 6 h of PDGF stimulation, while after longer stimulation, the expression levels decreased to become significantly downregulated at 72 h (Figure 2A).

We also analyzed the time course response to PDGF of OTUD6B-AS1 and OTUD6B in HC dermal Fb. In general, the effects of PDGF on the expression of OTUD6B-AS1 and OTUD6B in HC dermal Fb were similar to that observed in SSc dermal Fb (Figure 2B). These data suggest that PDGF strongly suppresses OTUD6B-AS1 and OTUD6B expression in dermal Fb, reflecting the expression pattern observed in SSc skin samples.

The effects after stimulation with other pro-fibrotic and pro-inflammatory cytokines were overall less consistent. OTUD6B-AS1 expression was slightly, but significantly upregulated after 72 h of TGFβ stimulation and significantly downregulated after 72 h of IL-4 stimulation. IL-4 slightly increased OTUD6B expression after 48 h of stimulation. However, we could not detect any strong and consistent effect after TGFβ, IL-4, and IL-13 stimulation (Supplementary Figures 2A,B) as observed for PDGF.

### OTUD6B-AS1 Knockdown Reduces Proliferation and Suppresses Apoptosis of Dermal Fb

**Figure 3:**
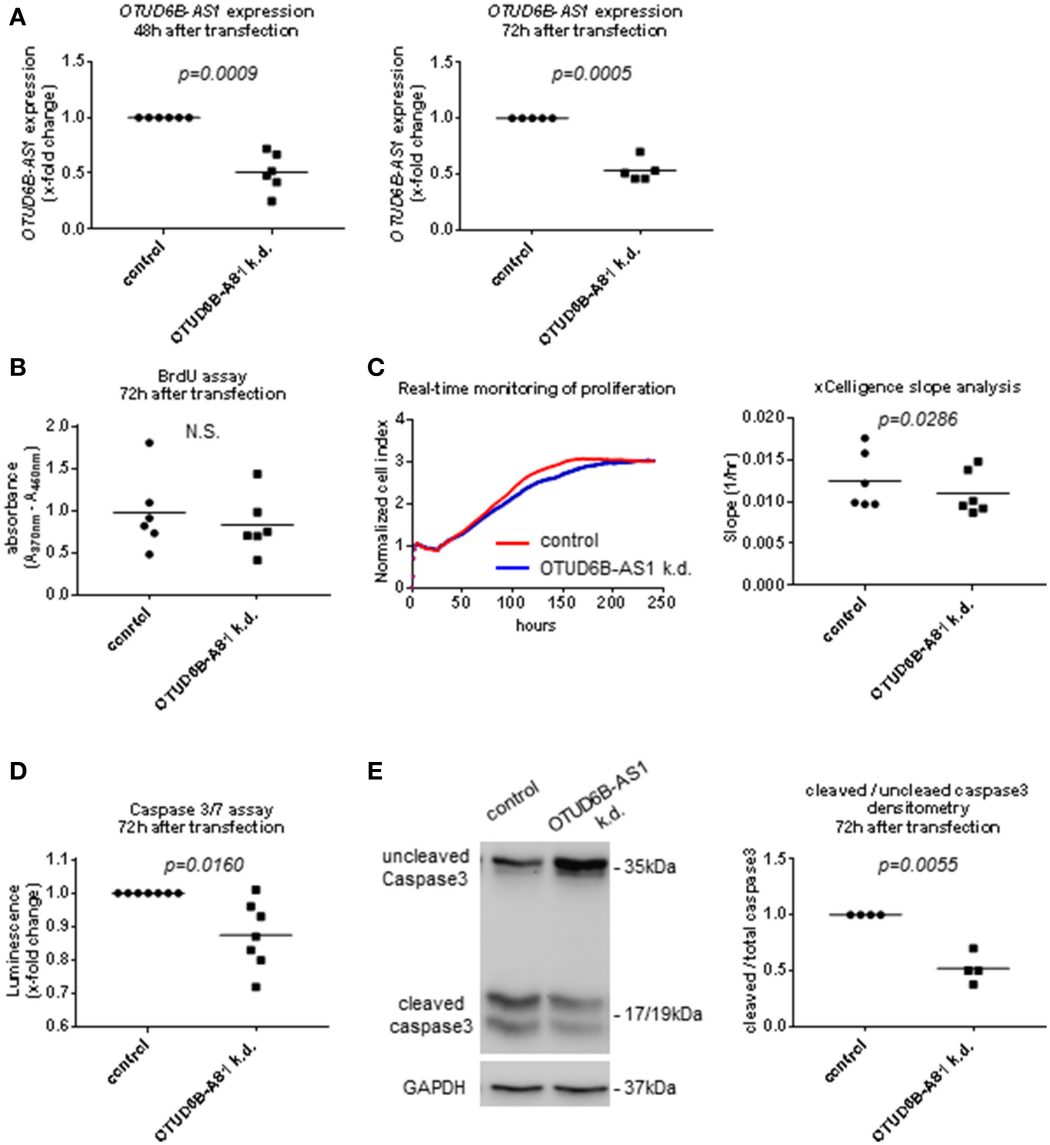
OTUD6B-AS1 knockdown in dermal fibroblasts (Fb): effects on proliferation and apoptosis. HC dermal Fb were transfected with locked nucleic acid antisense oligonucleotide (ASO) negative control or ASO targeting OTUD6B-AS1. **(A)** Efficacy of OTUD6B-AS1 knockdown 48 and 72 h after transfection in dermal Fb was confirmed by qPCR (*n* = 5–6). **(B)** BrdU cell proliferation assay performed 72 h after transfection (*n* = 6). **(C)** Real-time monitoring of cell proliferation analysis. Cell index (CI) was monitored every 5 min from 0 to 13 h, every 15 min from 13 to 25 h and every 30 min from 25 h until it reached plateau. CI was normalized at the time of transfection. Slope analysis was performed by RTCA software 2.0 (ACEA Biosciences) (*n* = 6, representative picture is shown). **(D)** Caspase 3/7 assay performed 72 h after transfection. Cells were treated by 1 μM staurosporin (STP, Sigma) for a period of 16 h to induce apoptosis (*n* = 7). **(E)** Western blot analysis of uncleaved and cleaved caspase 3 was performed 72 h after transfection. Cells were treated with 1 μM staurosporin (STP, Sigma) for a period of 16 h to induce apoptosis. GAPDH was used as a loading control. Densitometry analysis of cleaved caspase 3 was normalized to uncleaved caspase 3 (*n* = 4). Data are shown as single values and mean. Statistical analysis was performed by paired *t*-test. k.d, knockdown.

In order to further understand its function in the pathogenesis of SSc, we knocked down OTUD6B-AS1 using ASO (33). We used HC dermal Fb to mimic the downregulation seen in the SSc patients and to recapitulate the extent of OTUD6B-AS1 dysregulation effects in healthy cells. Successful knockdown of OTUD6B-AS1 was confirmed using qPCR at 48 and 72 h after transfection in HC dermal Fb (Figure 3A). First, we looked at effects of OTUD6B-AS1 knockdown on the expression of genes relevant for key processes of fibrosis. However, we could not detect significant differences in the gene expression of collagen 1α1 (COL1A1), fibronectin-1 (FN1) and alpha smooth muscle actin (α-SMA) (Supplementary Figure 3). These data indicate that OTUD6B-AS1 does not directly affect the expression of fibrotic genes.

Proliferation and apoptosis are also important processes in SSc pathogenesis (3). Thus, we assessed OTUD6B-AS1 function on dermal Fb proliferation using the BrdU assay and real-time monitoring of cell proliferation. Seventy-two hours after OTUD6B-AS1 knockdown, we observed a minor reduction in cell proliferation as detected by BrdU assay, however there was no statistically significant difference (Figure 3B). Real-time monitoring of cell proliferation showed that after more than 75 h of OTUD6B-AS1 knockdown, dermal Fb proliferated significantly slower than scrambled control treated Fb (Figure 3C).

We also assess apoptosis after OTUD6B-AS1 knockdown using Caspase 3/7 Glo assay® and cleaved caspase 3 WB analysis. Caspase 3/7 activity was slightly, but significantly downregulated 72 h after OTUD6B-AS1 knockdown (Figure 3D). Moreover, the ratio between cleaved and uncleaved caspase 3 was significantly reduced 72 h after OTUD6B-AS1 knockdown in dermal Fb (Figure 3E, Supplementary Figure 4A,B).

### OTUD6B-AS1 Knockdown Reduces Proliferation and Suppresses Apoptosis in HPASMC

**Figure 4:**
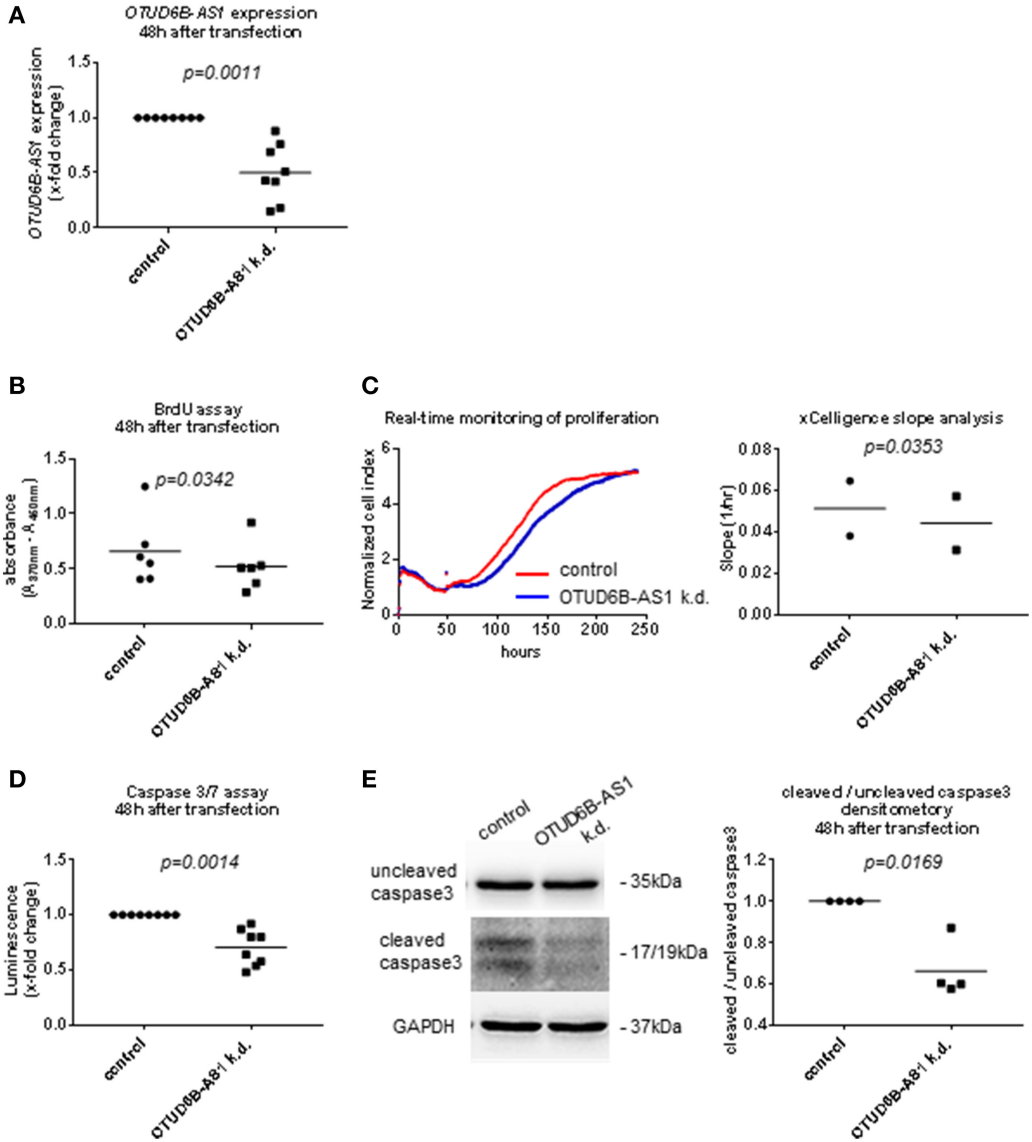
OTUD6B-AS1 knockdown in human pulmonary artery smooth muscle cells (HPASMC): effects on proliferation and apoptosis. HPASMC were transfected with locked nucleic acid antisense oligonucleotide (ASO) negative control or ASO targeting OTUD6B-AS1. **(A)** Efficacy of OTUD6B-AS1 knockdown 48 h after transfection in HPASMC was confirmed by qPCR. **(B)** BrdU cell proliferation assay performed 48 h after transfection (*n* = 6, biological replicates). **(C)** Real-time monitoring of cell proliferation. Cell index (CI) was monitored every 5 min from 0 to 13 h, every 15 min from 13 to 25 h and every 30 min from 25 h until it reached plateau. CI was normalized at the time of transfection. Slope analysis was performed by RTCA software 2.0 (ACEA Biosciences, *n* = 2, biological replicates, a representative picture is shown). **(D)** Caspase 3/7 assay performed 48 h after transfection. Cells were treated by 1 μM staurosporin for a period of 16 h to induce apoptosis (*n* = 8, technical replicates). **(E)** Western blot analysis of uncleaved and cleaved caspase 3 was performed 48 h after transfection. Cells were treated with 1 μM staurosporin (STP, Sigma) for a period of 16 h to induce apoptosis before cell collection. The same nitrocellulose membrane was used to detect uncleaved caspase 3 and cleaved caspase 3 with different exposure time for detection. GAPDH was used as a loading control. Densitometry analysis of cleaved caspase 3 was normalized to uncleaved caspase 3 (*n* = 4, biological replicates). Data are shown as single values and mean. Statistical analysis was performed by paired *t*-test. k.d, knockdown.

In the pathophysiology of SSc, cell cycle regulation, proliferation, and resistance to apoptosis is particularly important for the development of microvascular lesions, where vascular smooth muscle cells strongly proliferate resulting in vessel wall thickening and an occlusion of small arteries. The main clinical manifestation is PAH (3–5, 34). Therefore, we also performed OTUD6B-AS1 knockdown in HPASMC. OTUD6B-AS1 expression was significantly reduced in HPASMC at 48 h after transfection (Figure 4A, Supplementary Figure 4C–E). We also assessed proliferation and apoptosis after knockdown of OTUD6B-AS1 in HPASMC. HPASMC proliferation was significantly reduced already 48 h after transfection as revealed by BrdU assay (Figure 4B). Real-time monitoring of cell proliferation confirmed that HPASMC showed reduced proliferation earlier than dermal Fb and continued to proliferate significantly slower than scrambled control treated cells (Figure 4C). The apoptosis assay revealed that caspase 3/7 activity was significantly lower 48 h after OTUD6B-AS1 knockdown (Figure 4D), and the ratio of cleaved and uncleaved caspase 3 was significantly reduced 48 h after knockdown (Figure 4E) similar as for dermal Fb. These data suggest that OTUD6B-AS1 might be a regulator of proliferation and apoptosis not only for dermal Fb, but also for HPASMC.

### OTUD6B-AS1 Knockdown Increases CyclinD1 Expression in Dermal Fb and HPASMC

**Figure 5:**
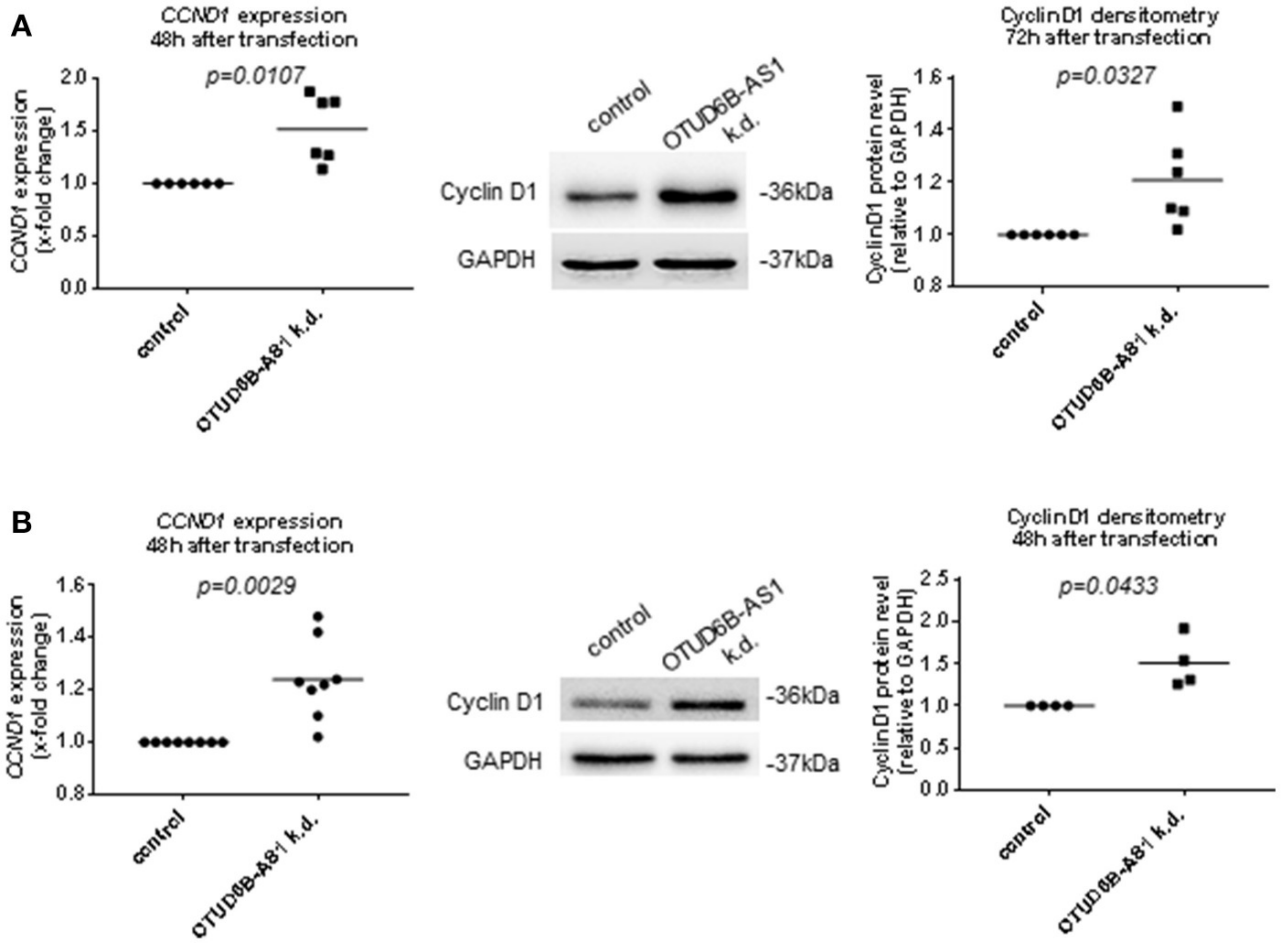
Cyclin D1 expression after OTUD6B-AS1 knockdown in dermal fibroblasts (Fb) or human pulmonary artery smooth muscle cells (HPASMC). HC dermal Fb or HPASMC were transfected with locked nucleic acid antisense oligonucleotide (ASO) negative control or ASO targeting OTUD6B-AS1. **(A)** mRNA level of cyclin D1 (CCND1) expression was measured 48 h after transfection (*n* = 6) and Cyclin D1 protein was measured 72 h after transfection (*n* = 6) in dermal Fb by Western blot. **(B)** mRNA level of cyclin D1 (CCND1) expression (*n* = 8, biological replicates) and Cyclin D1 protein (*n* = 4, biological replicates) was measured 48 h after transfection in HPASMC by Western blot. mRNA expression was normalized by GAPDH and RPLP0 and protein expression was normalized by GAPDH. Data are shown as single values and mean. Statistical analysis was performed by paired *t*-test. k.d, knockdown.

Cyclins are among the most important cell proliferation associated genes. They form active complexes with cyclin-dependent kinases and regulates cell cycle progression (35, 36).Thus, we analyzed the expression of the cell cycle regulators Cyclin D1, Cyclin D2, and pro-proliferative transcription factor MYC in dermal Fb after OTUD6B-AS1 knockdown. Cyclin D1 expression was significantly upregulated at mRNA level and protein level, respectively, 48 and 72 h after transfection (Figure 5A, Supplementary Figure 5). Cyclin D2 and MYC expression was unchanged both at the mRNA and protein level (Supplementary Figures 6A–D). We also analyzed additional pro-fibrotic and/or proinflammatory cytokines such as IL-6 and IGFBP3, and other cytokines that act on Fb to promote fibrotic responses, such as TGFB1 (37). However, after OTUD6B-AS1 knockdown IL-6, TGFB1, and IGFBP3 expression was unchanged (Supplementary Figure 7). Upregulation of Cyclin D1 was confirmed in an independent set of experiments using different two different anti-sense oligonucleotides targeting OTUD6B-AS1 (Supplementary Figure 8A).

As observed in dermal Fb, Cyclin D1 expression was significantly upregulated 48 h after OTUD6B-AS1 knockdown at the mRNA and at the protein level in HPASMC (Figure 5B, Supplementary Figure 8B). Cyclin D2 expression at the mRNA level was slightly upregulated, while MYC expression was unchanged (Supplementary Figure 9).

In addition, we analyzed the expression of the transcription factor E2F1, which is located downstream of Cyclin D1 and has been suggested as an apoptosis repressor in this context (38). However, we could not detect a consistent upregulation of the expression of E2F1, neither on the mRNA nor on the protein level in dermal Fb (Supplementary Figures 10A,B). These data suggest that OTUD6B-AS1 knockdown reduces cell proliferation and suppresses apoptosis in dermal Fb likely independent from E2F1.

Similarly, we did not detect any difference of E2F1 expression at the mRNA level and protein level 48 h after transfection in HPASMC (Supplementary Figures 10C,D).

All together, these results demonstrate that OTUD6B-AS1 targets Cyclin D1 and therefore it might be involved in cell proliferation and cell cycle regulation.

### OTUD6B-AS1 Knockdown Does Not Affect OTUD6B Expression

**Figure 6:**
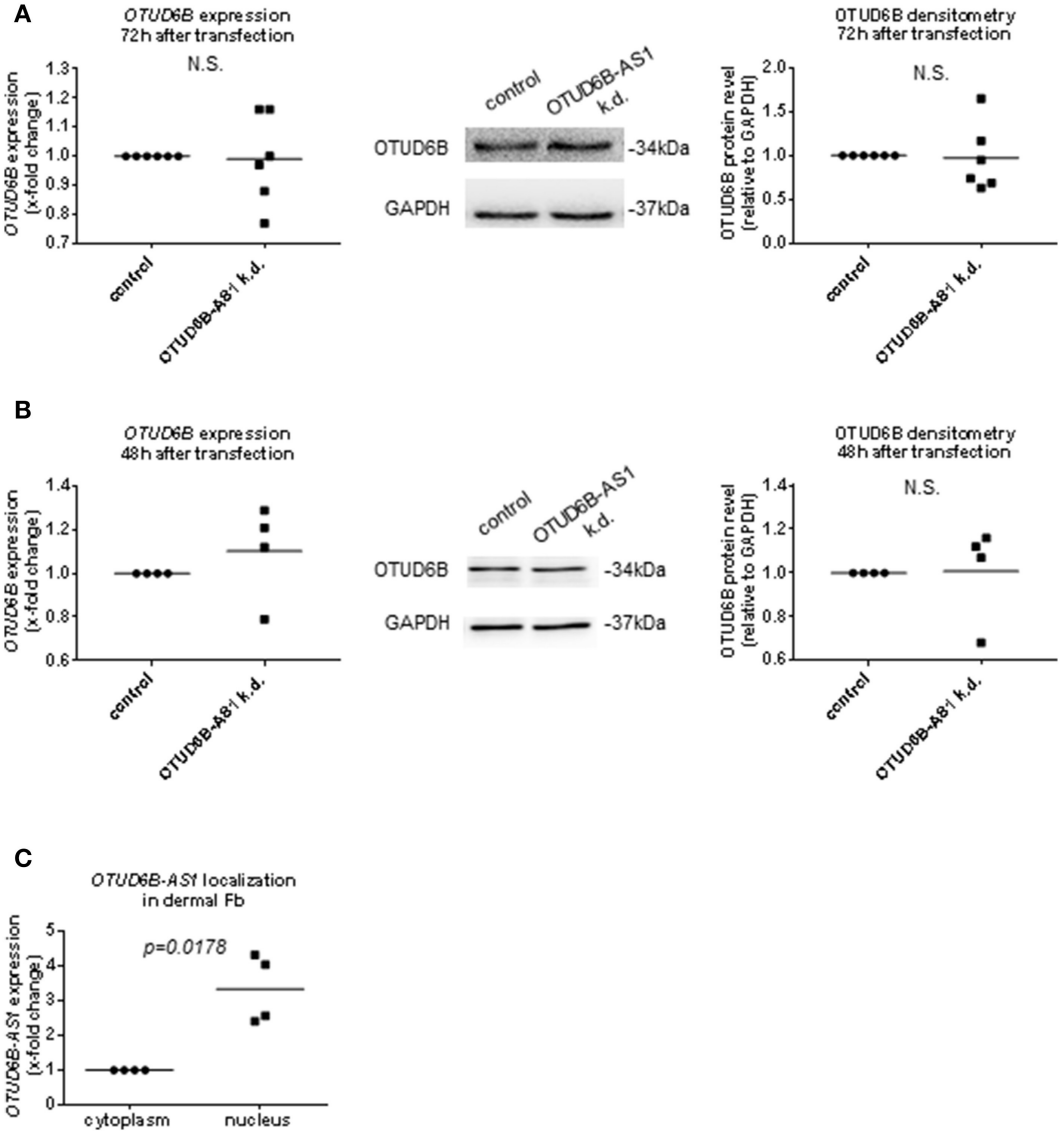
OTUD6B expression after OTUD6B-AS1 knockdown in dermal fibroblasts (Fb, **A**) or human pulmonary artery smooth muscle cells (HPASMC, **B**). HC dermal Fb or HPASMC were transfected with locked nucleic acid antisense oligonucleotide (ASO) negative control or with ASO targeting OTUD6B-AS1. **(A)** mRNA of total OTUD6B was measured 72 h after transfection (*n* = 5). OTUD6B protein was measured 72 h after transfection (*n* = 6) in dermal Fb by Western blot. **(B)** mRNA of total OTUD6B was measured 48 h after transfection (*n* = 4, biological replicates). OTUD6B protein expression was measured 48 h after transfection by Western blot (*n* = 4, biological replicates) in HPASMC. mRNA expression was normalized by GAPDH and RPLP0 and protein expression was normalized by GAPDH. **(C)** The subcellular localization of OTUD6B-AS1 in SSc dermal Fb was analyzed by qPCR (*n* = 4). Expression levels were normalized by GAPDH and RPLP0. Data are shown as single values and mean. Statistical analysis was performed by paired *t*-test. k.d, knockdown.

Regulation of corresponding sense gene expression is the most common mechanism of action of antisense transcripts (12–14, 39). Therefore, we analyzed mRNA expression and protein expression of OTUD6B after OTUD6B-AS1 knockdown. Total OTUD6B expression at the mRNA and protein level was unchanged after OTUD6B-AS1 knockdown in dermal Fb (Figure 6A, Supplementary Figure 8A and Supplementary Figure 11A,B). Similarly, in HPASMC, OTUD6B expression after OTUD6B-AS1 knockdown was not changed (Figure 6B, Supplementary Figure 8B and Supplementary Figure 11C,D).

During processing, lncRNAs are transported into the cytoplasm or remain in the nucleus (8, 40) to exert their function. Thus, the location of OTUD6B-AS1 can further give hints about its mechanism of action. By using cell fractionation experiments, we found that OTUD6B-AS1 was mainly localized in the nucleus of dermal SSc Fb (Figure 6C).

Overall, these data indicate that OTUD6B-AS1 knockdown does not affect expression of its sense gene OTUD6B in dermal Fb and HPASMC. The effects on target gene Cyclin D1, apoptosis, and proliferation are likely to occur in the nucleus and might be due to OTUD6B-AS1 *trans* function rather than influencing OTUD6B sense gene expression.

## Discussion

AS lncRNAs have been identified as crucial players in the pathogenesis of different diseases such as cancer, however little is known in fibrotic disorders including SSc. In this study, we investigated the role of OTUD6B-AS1 in the pathogenesis of SSc.

First, our additional analyses on the expression pattern showed that the downregulation of OTUD6B-AS1 and OTUD6B mRNA was seen particularly in clinically involved, fibrotic skin biopsies of SSc patients. This finding might give hints on their pathophysiological role. However, the cohorts studied in these experiments were rather limited in patient numbers and thus, our findings should be confirmed in additional, larger cohorts. In addition, due to the limited availability of protein from skin biopsies, the downregulation of OTUD6B should be confirmed by Western blots from biopsies of involved skin in additional studies. We then analyzed the effects of pro-fibrotic and pro-inflammatory cytokines on the expression of OTUD6B-AS1 and OTUD6B. Pro-inflammatory cytokines are playing a major role in the pathogenesis of SSc, in particular in early, but also in later stages of the disease (41). We could show that the expression of OTUD6B-AS1 and OTUD6B was significantly downregulated after PDGF simulation in dermal Fb. PDGF plays an important role in fibrosis and regulation of inflammation. PDGFs are primary mitogens and chemo-attractants for mesenchymal cells and they are secreted from platelets, macrophages, fibroblasts, and endothelial cells (1). PDGF and PDGF receptor β are highly expressed in SSc skin (42). Activated microvascular pericytes express more PDGFβ receptors in early SSc patients than normal control or late-stage SSc patients (43). Transgenic mice with constitutively activated PDGF receptor α develop multiple organ fibrosis, including skin fibrosis (44). Our data indicated that PDGF is an important regulator of OTUD6B-AS1 and OTUD6B expression in dermal Fb.

In order to identify the functional role of OTUD6B-AS1, we performed knockdown experiments of OTUD6B-AS1 using ASO in HC dermal Fb and HPASMC. Dermal Fb from SSc patients display increased proliferation in culture (45), and it has been demonstrated that proliferation of vascular smooth muscle cells is a key feature of the proliferative microangiopathy characteristic for the vascular manifestations of SSc (46). Cyclin D1 was identified as an OTUD6B-AS1 target in both dermal Fb and HPASMC. Cyclin D1 is a well-characterized cell cycle regulator and oncogene (36, 47, 48). Surprisingly, after OTUD6B-AS1 knockdown, we observed increased cyclin D1 expression, but reduced cell proliferation. Cyclin D1 expression levels are subjected to change throughout the cell cycle. Specifically, a high level of cyclin D1 expression is required for G1 phase. However, cyclin D1 decreases during the S phase for efficient DNA synthesis and increase again in G2 phase to continue proliferation (49, 50). This phenomenon was observed in different cell types including fibroblasts (50). Moreover, Cyclin D1 also has an inhibitory function on DNA synthesis via binding with proliferation cell nuclear antigen (PCNA) (51). Overexpression of cyclin D1 in human Fb prevents DNA repair and prevents cells from entering in S phase (52). This mechanism could explain the reduction in proliferation that we observed in dermal Fb and HPASMC after OTUD6B-AS1 knockdown. Thus, while the OTUD6B-AS1 knockdown favored apoptosis resistance (see below), which is supporting the pathogenesis of SSc, an inhibition of proliferation would have to be interpreted as a compensatory mechanisms. We did use HC cells for these experiments to better mimic the downregulation found in SSc skin. In further studies, these effects should be confirmed in primary SSc cells, to exclude influence of SSc specific pathway alterations on our results.

We showed that OTUD6B-AS1 knockdown suppresses apoptosis in dermal Fb and HPASMC. Fibrosis is a predominant phenomenon in SSc. Fibrotic tissue is characterized by apoptosis resistant myofibroblasts that arise from resident fibroblasts and other cellular sources (3–5). The mechanisms of the resistance to apoptosis are largely unknown. We have identified regulation of cyclin D1 by OTUD6B-AS1 in dermal Fb as a possible novel contributor to apoptosis resistance in SSc. Accordingly, it has been reported that D-type cyclins (D1, D2, and D3) can repress apoptosis in hematopoietic cells. Previous studies showed that this effect of cyclins could be mediated via the transcription factor E2F1, which in turn regulates the expression of Fas and Fas ligand (38). However, we could not detect consistent changes in the expression of E2F1 after knockdown of OTUD6B-AS1. With our experimental set-up, we cannot completely exclude that there are subtle changes of E2F1 and it downstream targets Fas and Fas ligand at other time points, but other, E2F1 independent mechanisms are much more likely. Knockdown of cyclin D1 could provide further insight into downstream targets mediating the apoptosis resistance in SSc fibroblasts.

We also investigated the relationship between the expression of OTUD6B-AS1 and sense gene OTUD6B. Our data showed that PDGF stimulation downregulated the expression of both OTUD6B-AS1 and OTUD6B, initially pointing to linked mechanisms of action. However, while the majority of anti-sense genes mediate their effects via direct interaction with the sense gene, we did not detect any change of the expression of OTUD6B on the RNA and protein level after OTUD6B-AS1 knockdown. Recent investigations showed that in some circumstances, the mature AS transcript does not influence sense gene expression and the AS transcription itself is required for sense gene expression (14, 53). This means, silencing AS transcript using ASO does not necessarily affect sense gene expression. In our experiments, we used ASO to reduce mature OTUD6B-AS1 levels and this might explain why OTUD6B expression was not affected. On the other hand, our data suggest that OTUD6B-AS1 has an independent mechanism from its sense gene.

Taken together, we provide the first evidence for a functional role of OTUD6B-AS1 in two cell types that play a major role in SSc. We showed that the expression of OTUD6B-AS1 and OTUD6B was significantly downregulated in dermal Fb after PDGF stimulation. OTUD6B-AS1 knockdown experiments revealed that OTUD6B-AS1 controls proliferation and apoptosis of dermal Fb and HPASMC through the regulation of cyclin D1 expression. The OTUD6B-AS1 mode of action was independent of its sense gene OTUD6B. These results suggest that OTUD6B-AS1 downregulation might promote apoptosis resistance of Fb and HPASMC contributing to cell dysregulation on the pathophysiology of SSc.

## Ethics Statement

This study was carried out in accordance with the recommendations of World Medical Association Declaration of Helsinki, ICH-GCP guidelines, or ISO 14155. The study was approved by the Ethics Committee of the Canton of Zurich (approved ethical applications KEK-ZH 515, PB-2016-02014, and KEK-Nr. 2018-01873). All subjects gave written informed consent in accordance with the Declaration of Helsinki.

## Author Contributions

MT, EP, and OD: study conception and design. MT, EP, MF-B, AK, MW, SA, MC, TM, JdV-B, and FK: acquisition of data. MT, EP, MF-B, AJ, TM, JdV-B, TH, FK, GK, and OD: analysis and interpretation of data. MT, EP, and OD: drafting and revising the article. All authors have seen and approved the manuscript and its content and are aware of the responsibilities connected to authorship.

### Conflict of Interest Statement

OD has consultancy relationship with Actelion, Bayer, BiogenIdec, Boehringer Ingelheim, ChemomAb, espeRare foundation, Genentech/Roche, GSK, Inventiva, Italfarmaco, Lilly, medac, MedImmune, Mitsubishi Tanabe Pharma, Pharmacyclics, Novartis, Pfizer, Sanofi, Sinoxa, and UCB in the area of potential treatments of scleroderma and its complications. OD has received research funding from Actelion, Bayer, Boehringer Ingelheim, Mitsubishi Tanabe Pharma, and Roche in the area of potential treatments of scleroderma and its complications. In addition, OD has a patent mir-29 for the treatment of systemic sclerosis licensed. The remaining authors declare that the research was conducted in the absence of any commercial or financial relationships that could be construed as a potential conflict of interest.

## References

[1] Trojanowska M. Role of PDGF in fibrotic diseases and systemic sclerosis. Rheumatology. (2008) 47(Suppl. 5):v2–4. 10.1093/rheumatology/ken265 18784131

[2] Lafyatis R. Transforming growth factor β-at the centre of systemic sclerosis. Nat Rev Rheumatol. (2014) 10:706–19. 10.1038/nrrheum.2014.137 25136781

[3] Allanore Y Simms R Distler O Trojanowska M Pope J Denton CP Systemic sclerosis. Nat Rev Dis Primers. (2015) 1:15002 10.1038/nrdp.2015.2 27189141

[4] Distler JH Feghali-Bostwick C Soare A Asano Y Distler O Abraham DJ. Review: frontiers of antifibrotic therapy in systemic sclerosis. Arthritis Rheumatol. (2017) 69:257–67. 10.1002/art.39865 27636741

[5] Khanna D Distler JH Sandner P Distler O Emerging strategies for treatment of systemic sclerosis. J Scleroderm Relat Dis. (2016) 1:186–93. 10.5301/jsrd.5000207

[6] Devaux Y Zangrando J Schroen B Creemers EE Pedrazzini T Chang CP Long noncoding RNAs in cardiac development and aging. Nat Rev Cardiol. (2015) 12:415–25. 10.1038/nrcardio.2015.55 25855606

[7] Ozsolak F Kapranov P Foissac S Kim SW Fishilevich E Monaghan AP. Comprehensive polyadenylation site maps in yeast and human reveal pervasive alternative polyadenylation. Cell. (2010) 143:1018–29. 10.1016/j.cell.2010.11.020 21145465 PMC3022516

[8] Derrien T Johnson R Bussotti G Tanzer A Djebali S Tilgner H. The GENCODE v7 catalog of human long noncoding RNAs: analysis of their gene structure, evolution, and expression. Genome Res. (2012) 22:1775–89. 10.1101/gr.132159.111 22955988 PMC3431493

[9] Fatica A Bozzoni I. Long non-coding RNAs: new players in cell differentiation and development. Nat Rev Genet. (2014) 15:7–21. 10.1038/nrg3606 24296535

[10] Clarl BS Blackshaw S Long non-coding RNA-dependent transcriptional regulation in neuronal development and disease. Front Genet. (2014) 6:164 10.3389/fgene.2014.00164 PMC4047558 24936207

[11] Kathuria H Millien G McNally L Gower AC Tagne JB Cao Y. NKX2-1-AS1 negatively regulates CD274/PD-L1, cell-cell interaction genes, and limits human lung carcinoma cell migration. Sci Rep. (2018) 8:14418. 10.1038/s41598-018-32793-5 30258080 PMC6158174

[12] Pelechano V Steinmetz LM. Gene regulation by antisense transcription. Nat Rev Genet. (2013) 14:880–93. 10.1038/nrg3594 24217315

[13] Villegas VE Zaphiropoulos PG. Neighboring Gene regulation by antisense long non-coding RNAs. Int J Mol Sci. (2015) 16:3251–66. 10.3390/ijms16023251 25654223 PMC4346893

[14] Faghihi MA Wahlestedt C. Regulatory roles of natural antisense transcripts. Nat Rev Mol Cell Biol. (2009) 10:637–43. 10.1038/nrm2738 19638999 PMC2850559

[15] Gupta RA Shah N Wang KC Kim J Horlings HM Wong DJ. Long non-coding RNA HOTAIR reprograms chromatin state to promote cancer. Nature. (2010) 464:1071–6. 10.1038/nature08975 20393566 PMC3049919

[16] Yap KL Li S Muñoz-Cabello AM Raguz S Zeng L Mujtaba S. Molecular interplay of the noncoding RNA ANRIL and methylated histone H3 lysine 27 by polycomb CBX7 in transcriptional silencing of INK4a. Mol Cell. (2010) 38:662–74. 10.1016/j.molcel.2010.03.021 20541999 PMC2886305

[17] Mondal T Juvvuna PK Kirkeby A Mitra S Kosalai ST Traxler L. Sense-antisense lncRNA pair encoded by locus 6p22.3 determines neuroblastoma susceptibility via the USP36-CHD7-SOX9 regulatory axis. Cancer Cell. (2018) 33:417–34.e7. 10.1016/j.ccell.2018.01.020 29533783

[18] Lorenzen JM Thum T. Long noncoding RNAs in kidney and cardiovascular diseases. Nat Rev Nephrol. (2016) 12:360–73. 10.1038/nrneph.2016.51 27140855

[19] Faghihi MA Modarresi F Khalil AM Wood DE Sahagan BG Morgan TE. Expression of a noncoding RNA is elevated in Alzheimer's disease and drives rapid feed-forward regulation of beta-secretase. Nat Med. (2008) 14:723–30. 10.1038/nm1784 18587408 PMC2826895

[20] Wang Z Jinnin M Nakamura K Harada M Kudo H Nakayama W. Long non-coding RNA TSIX is upregulated in scleroderma dermal fibroblasts and controls collagen mRNA stabilization. Exp Dermatol. (2016) 25:131–6. 10.1111/exd.12900 26566700

[21] Nong Q Li S Wu Y Liu D. LncRNA COL1A2-AS1 inhibits the scar fibroblasts proliferation via regulating miR-21/Smad7 pathway. Biochem Biophys Res Commun. (2018) 495:319–24. 10.1016/j.bbrc.2017.11.027 29117538

[22] Messemaker TC Chadli L Cai G Goelela VS Boonstra M Dorjée AL. Antisense long non-coding RNAs are deregulated in skin tissue of patients with systemic sclerosis. J Invest Dermatol. (2018) 138:826–35. 10.1016/j.jid.2017.09.053 29179949

[23] Clague MJ Barsukov I Coulson JM Liu H Rigden DJ Urbé S. Deubiquitylases from genes to organism. Physiol Rev. (2013) 93:1289–315. 10.1152/physrev.00002.2013 23899565

[24] Bhattacharya S Ghosh MK. Cell death and deubiquitinases: perspectives in cancer. Biomed Res Int. (2014) 2014:435197. 10.1155/2014/435197 25121098 PMC4119901

[25] Xu Z Zheng Y Zhu Y Kong X Hu L. Evidence for OTUD-6B participation in B lymphocytes cell cycle after cytokine stimulation. PLoS ONE. (2011) 6:e14514. 10.1371/journal.pone.0014514 21267069 PMC3022568

[26] Sobol A Askonas C Alani S Weber MJ Ananthanarayanan V Osipo C. Deubiquitinase OTUD6B isoforms are important regulators of growth and proliferation. Mol Cancer Res. (2017) 15:117–27. 10.1158/1541-7786.MCR-16-0281-T 27864334 PMC5290186

[27] Santiago-Sim T Burrage LC Ebstein F Tokita MJ Miller M Bi W. Biallelic variants in OTUD6B cause an intellectual disability syndrome associated with seizures and dysmorphic features. Am J Hum Genet. (2017) 100:676–88. 10.1016/j.ajhg.2017.03.001 28343629 PMC5384096

[28] LeRoy EC Black C Fleischmajer R Jablonska S Krieg T Medsger TA Jr. Scleroderma (systemic sclerosis): classification, subsets and pathogenesis. J Rheumatol. (1988) 15:202–5. 3361530

[29] Gagnon KT Li L Janowski BA Corey DR. Analysis of nuclear RNA interference in human cells by subcellular fractionation and Argonaute loading. Nat Protoc. (2014) 9:2045–60. 10.1038/nprot.2014.135 25079428 PMC4251768

[30] Schmittgen TD Livak KJ. Analyzing real-time PCR data by the comparative C(T) method. Nat Protoc. (2008) 3:1101–8. 10.1038/nprot.2008.73 18546601

[31] Tamaoki T Nomoto H Takahashi I Kato Y Morimoto M Tomita F. Staurosporine, a potent inhibitor of phospholipid/Ca^++^ dependent protein kinase. Biochem Biophys Res Commun. (1986) 135:397–402. 3457562 10.1016/0006-291x(86)90008-2

[32] Belmokhtar CA Torriglia A Counis MF Courtois Y Jacquemin-Sablon A Ségal-Bendirdjian E Nuclear translocation of a leukocyte elastase inhibitor/elastase complex during staurosporine-induced apoptosis: role in the generation of nuclear L-DNase II activity. Exp Cell Res. (2000) 25:99–109. 10.1006/excr.1999.4737 10623470

[33] Kole R Krainer AR Altman S. RNA therapeutics: beyond RNA interference and antisense oligonucleotides. Nat Rev Drug Discov. (2012) 11:125–40. 10.1038/nrd3625 22262036 PMC4743652

[34] Gilbane AJ Denton CP Holmes AM. Scleroderma pathogenesis: a pivotal role for fibroblasts as effector cells. Arthritis Res Ther. (2013) 15:215. 10.1186/ar4230 23796020 PMC4060542

[35] Malumbres M Barbacid M. Cell cycle, CDKs and cancer: a changing paradigm. Nat Rev Cancer. (2009) 9:153–66. 10.1038/nrc2602 19238148

[36] Sherr CJ. D-type cyclins. Trends Biochem Sci. (1995) 20:187–90. 7610482 10.1016/s0968-0004(00)89005-2

[37] Kendall RT Feghali-Bostwick CA. Fibroblasts in fibrosis: novel roles and mediators. Front Pharmacol. (2014) 5:123. 10.3389/fphar.2014.00123. 24904424 PMC4034148

[38] Choi YJ Saez B Anders L Hydbring P Stefano J Bacon NA. D-cyclins repress apoptosis in hematopoietic cells by controlling death receptor Fas and its ligand FasL. Dev Cell. (2014) 30:255–67. 10.1016/j.devcel.2014.06.015 25087893 PMC4134362

[39] Katayama S Tomaru Y Kasukawa T Waki K Nakanishi M Nakamura M. Antisense transcription in the mammalian transcriptome. Science. (2005) 309:1564–6. 10.1126/science.1112009 16141073

[40] Thum T Condorelli G. Long noncoding RNAs and microRNAs in cardiovascular pathophysiology. Circ Res. (2015) 116:751–62. 10.1161/CIRCRESAHA.116.303549 25677521

[41] Distler O Pap T Kowal-Bielecka O Meyringer R Guiducci S Landthaler M. Overexpression of monocyte chemoattractant protein 1 in systemic sclerosis: role of platelet-derived growth factor and effects on monocyte chemotaxis and collagen synthesis. Arthritis Rheum. (2001) 44:2665–78. 10.1002/1529-0131(200111)44:11<2665::AID-ART446>3.0.CO;2-S 11710722

[42] Klareskog L Gustafsson R Scheynius A Hällgren R. Increased expression of platelet-derived growth factor type B receptors in the skin of patients with systemic sclerosis. Arthritis Rheum. (1990)33:1534–41. 2171541 10.1002/art.1780331011

[43] Rajkumar VS Sundberg C Abraham DJ Rubin K Black CM. Activation of microvascular pericytes in autoimmune Raynaud's phenomenon and systemic sclerosis. Arthritis Rheum. (1999) 42:930–41. 10323448 10.1002/1529-0131(199905)42:5<930::AID-ANR11>3.0.CO;2-1

[44] Olson LE Soriano P. Increased PDGFRalpha activation disrupts connective tissue development and drives systemic fibrosis. Dev Cell. (2009) 16:303–13. 10.1016/j.devcel.2008.12.003 19217431 PMC2664622

[45] Santiago B Galindo M Rivero M Pablos JL. Decreased susceptibility to Fas-induced apoptosis of systemic sclerosis dermal fibroblasts. Arthritis Rheum. (2001) 44:1667–76. 10.1002/1529-0131(200107)44:7<1667::AID-ART291>3.0.CO;2-Y 11465719

[46] Trojanowska M. Cellular and molecular aspects of vascular dysfunction in systemic sclerosis. Nat Rev Rheumatol. (2010) 6:453–60. 10.1038/nrrheum.2010.102 20585340 PMC3824624

[47] Qie S Diehl JA. Cyclin D1, cancer progression, and opportunities in cancer treatment. J Mol Med. (2016) 94:1313–26. 10.1007/s00109-016-1475-3 27695879 PMC5145738

[48] Santarius T Shipley J Brewer D Stratton MR Cooper CS. A census of amplified and overexpressed human cancer genes. Nat Rev Cancer. (2010) 10:59–64. 10.1038/nrc2771 20029424

[49] Stacey DW. Cyclin D1 serves as a cell cycle regulatory switch in actively proliferating cells. Curr Opin Cell Biol. (2003) 15:158–63. 10.1016/S0955-0674(03)00008-5 12648671

[50] Yang K Hitomi M Stacey DW. Variations in cyclin D1 levels through the cell cycle determine the proliferative fate of a cell. Cell Div. (2016) 1:32. 10.1186/1747-1028-1-32 17176475 PMC1769361

[51] Fukami-Kobayashi J Mitsui Y Cyclin D1 inhibits cell proliferation through binding to PCNA and cdk2. Exp Cell Res. (1999) 46:338–47. 10.1006/excr.1998.4306 9925749

[52] Pagano M Theodoras AM Tam SW Draetta GF. Cyclin D1-mediated inhibition of repair and replicative DNA synthesis in human fibroblasts. Genes Dev. (1994) 8:1627–39. 7958844 10.1101/gad.8.14.1627

[53] Anderson KM Anderson DM McAnally JR Shelton JM Bassel-Duby R Olson EN. Transcription of the non-coding RNA upperhand controls Hand2 expression and heart development. Nature. (2016) 539:433–6. 10.1038/nature20128 27783597 PMC5261552

