# Supplementary material for: OTUD6B-AS1 Might Be a Novel Regulator of Apoptosis in Systemic Sclerosis
Source: Front Immunol. 2019 May 17;10:1100. doi: 10.3389/fimmu.2019.01100 (PMC6533854; doi:10.3389/fimmu.2019.01100)
Supplement: Supplementary file 1 [file Presentation_1.pptx]

## Slide 1
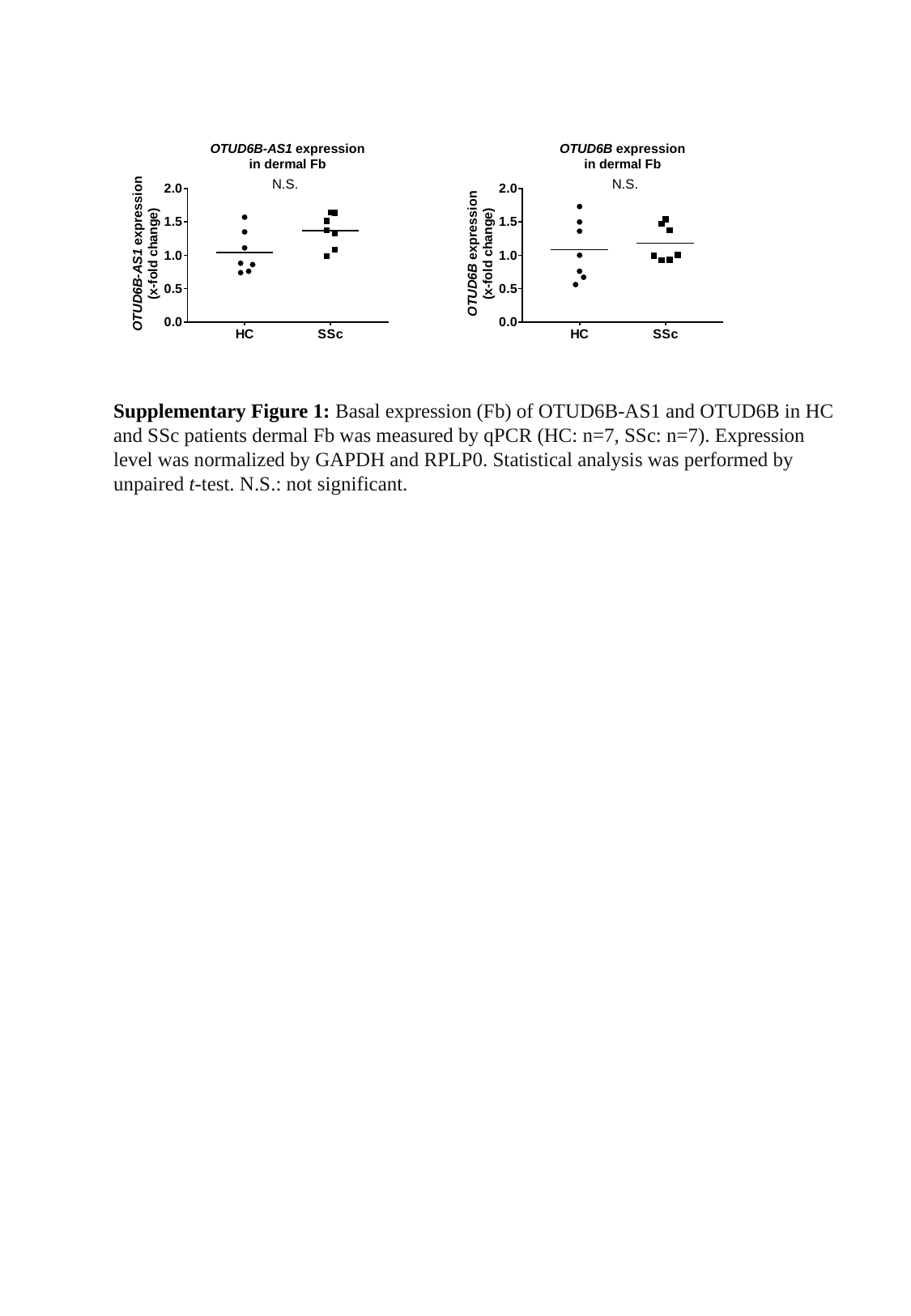

N.S.
N.S.
Supplementary Figure 1: Basal expression (Fb) of OTUD6B-AS1 and OTUD6B in HC and SSc patients dermal Fb was measured by qPCR (HC: n=7, SSc: n=7). Expression level was normalized by GAPDH and RPLP0. Statistical analysis was performed by unpaired t-test. N.S.: not significant.

## Slide 2
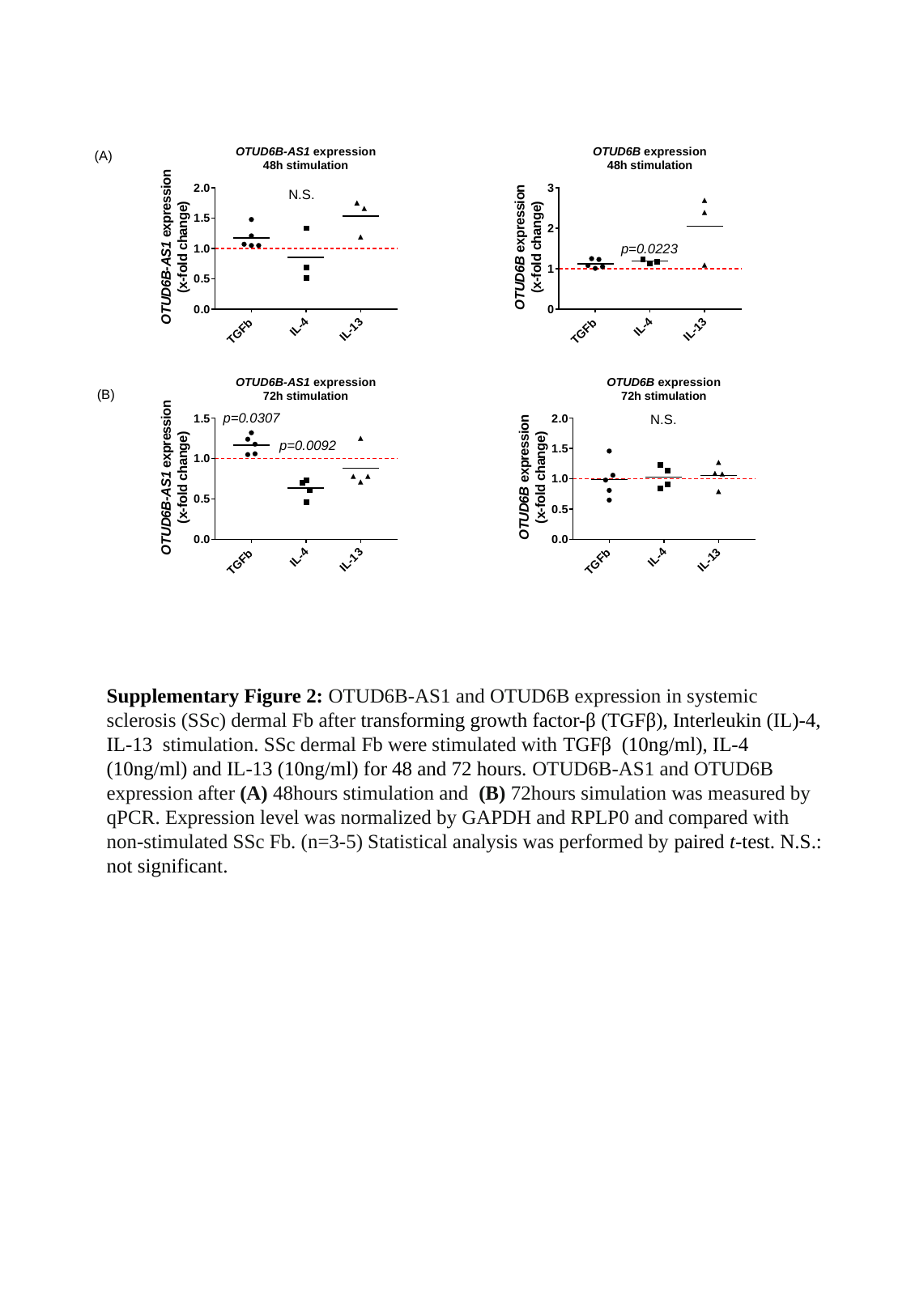

(A)
N.S.
p=0.0223
N.S.
(B)
p=0.0307
p=0.0092
Supplementary Figure 2: OTUD6B-AS1 and OTUD6B expression in systemic sclerosis (SSc) dermal Fb after transforming growth factor-β (TGFβ), Interleukin (IL)-4, IL-13 stimulation. SSc dermal Fb were stimulated with TGFβ (10ng/ml), IL-4 (10ng/ml) and IL-13 (10ng/ml) for 48 and 72 hours. OTUD6B-AS1 and OTUD6B expression after (A) 48hours stimulation and (B) 72hours simulation was measured by qPCR. Expression level was normalized by GAPDH and RPLP0 and compared with non-stimulated SSc Fb. (n=3-5) Statistical analysis was performed by paired t-test. N.S.: not significant.

## Slide 3
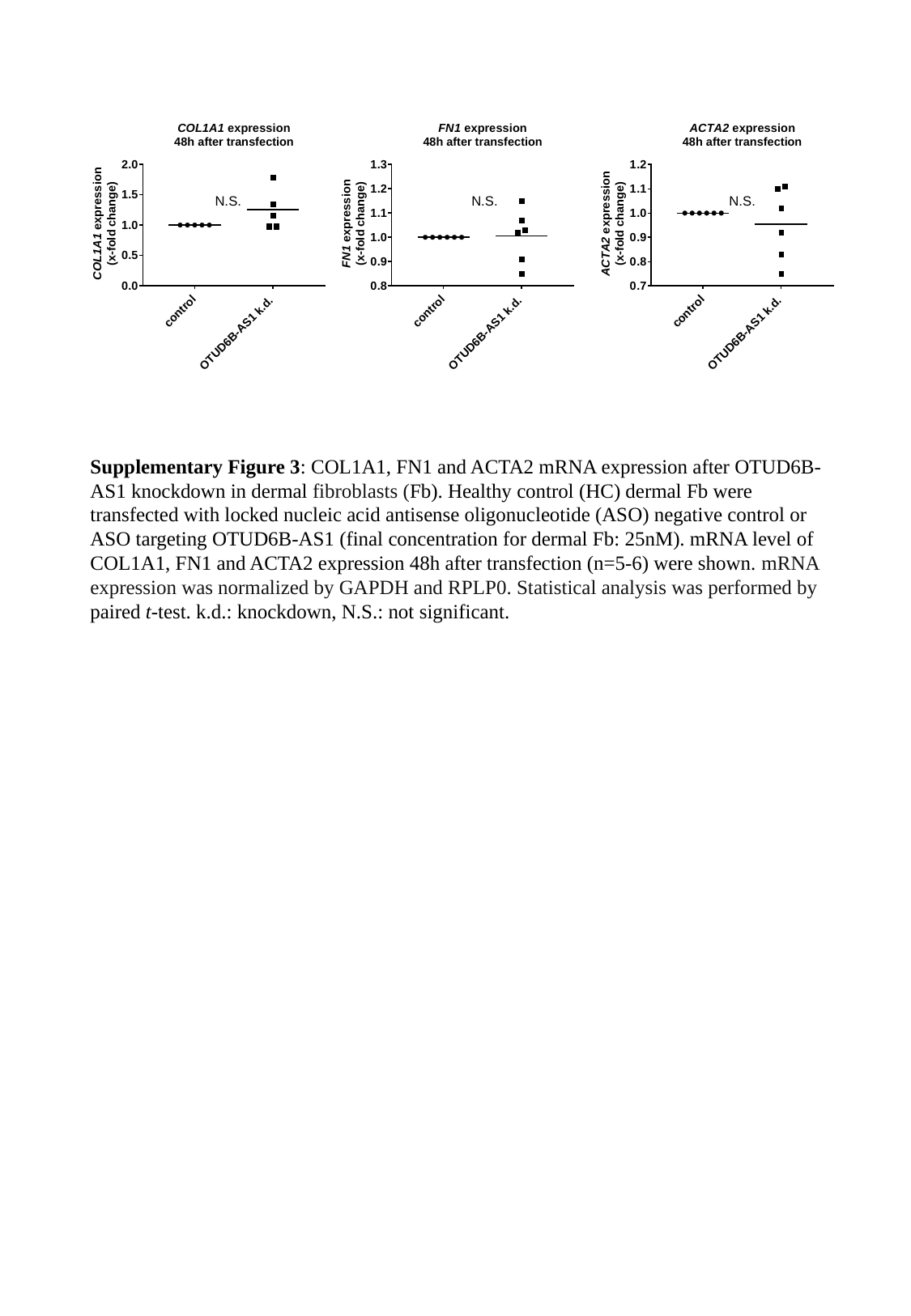

N.S.
N.S.
N.S.
Supplementary Figure 3: COL1A1, FN1 and ACTA2 mRNA expression after OTUD6B-AS1 knockdown in dermal fibroblasts (Fb). Healthy control (HC) dermal Fb were transfected with locked nucleic acid antisense oligonucleotide (ASO) negative control or ASO targeting OTUD6B-AS1 (final concentration for dermal Fb: 25nM). mRNA level of COL1A1, FN1 and ACTA2 expression 48h after transfection (n=5-6) were shown. mRNA expression was normalized by GAPDH and RPLP0. Statistical analysis was performed by paired t-test. k.d.: knockdown, N.S.: not significant.

## Slide 4
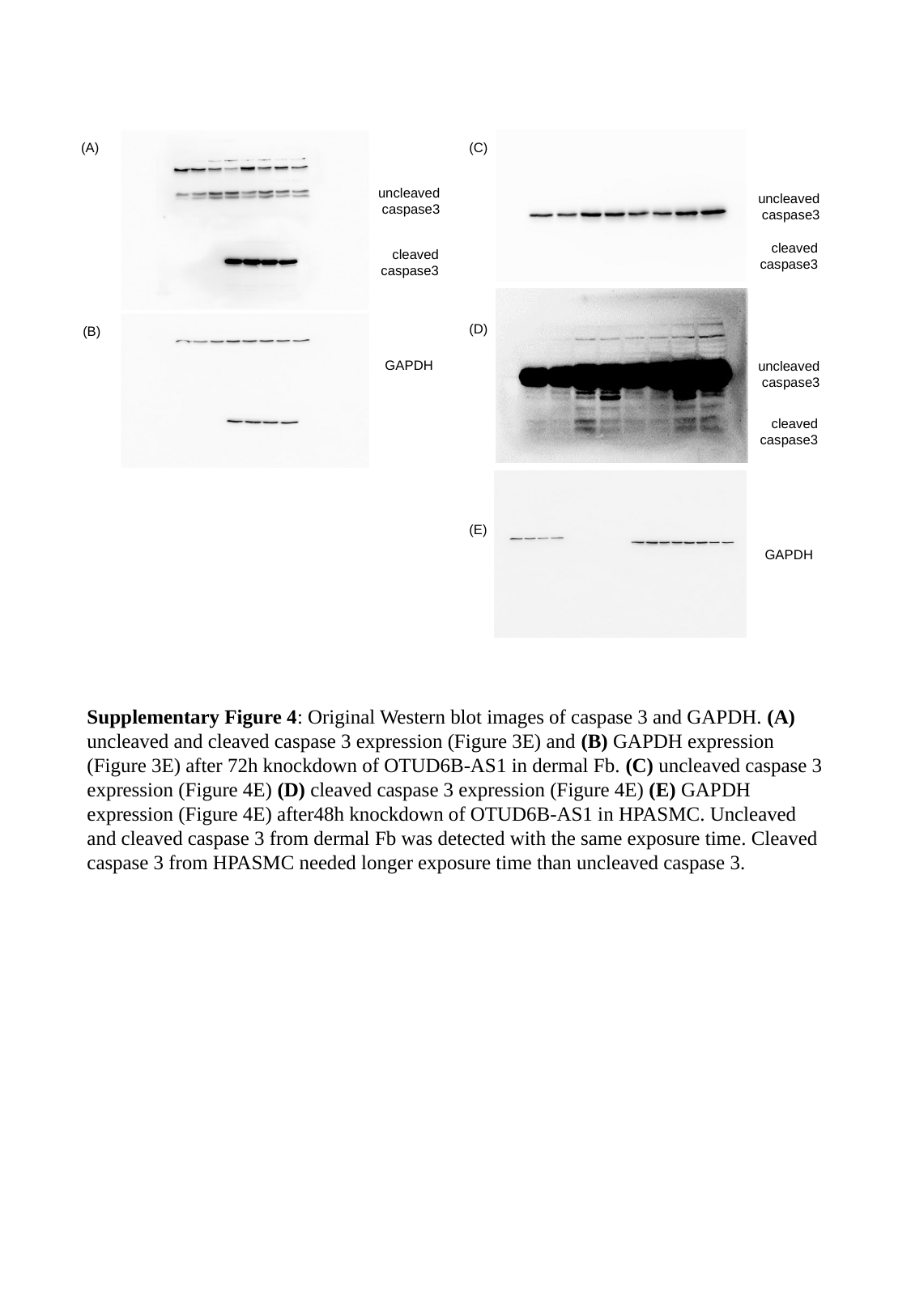

(A)
(C)
uncleaved
caspase3
uncleaved
caspase3
cleaved
caspase3
cleaved
caspase3
(D)
(B)
GAPDH
uncleaved
caspase3
cleaved
caspase3
(E)
GAPDH
Supplementary Figure 4: Original Western blot images of caspase 3 and GAPDH. (A) uncleaved and cleaved caspase 3 expression (Figure 3E) and (B) GAPDH expression (Figure 3E) after 72h knockdown of OTUD6B-AS1 in dermal Fb. (C) uncleaved caspase 3 expression (Figure 4E) (D) cleaved caspase 3 expression (Figure 4E) (E) GAPDH expression (Figure 4E) after48h knockdown of OTUD6B-AS1 in HPASMC. Uncleaved and cleaved caspase 3 from dermal Fb was detected with the same exposure time. Cleaved caspase 3 from HPASMC needed longer exposure time than uncleaved caspase 3.

## Slide 5
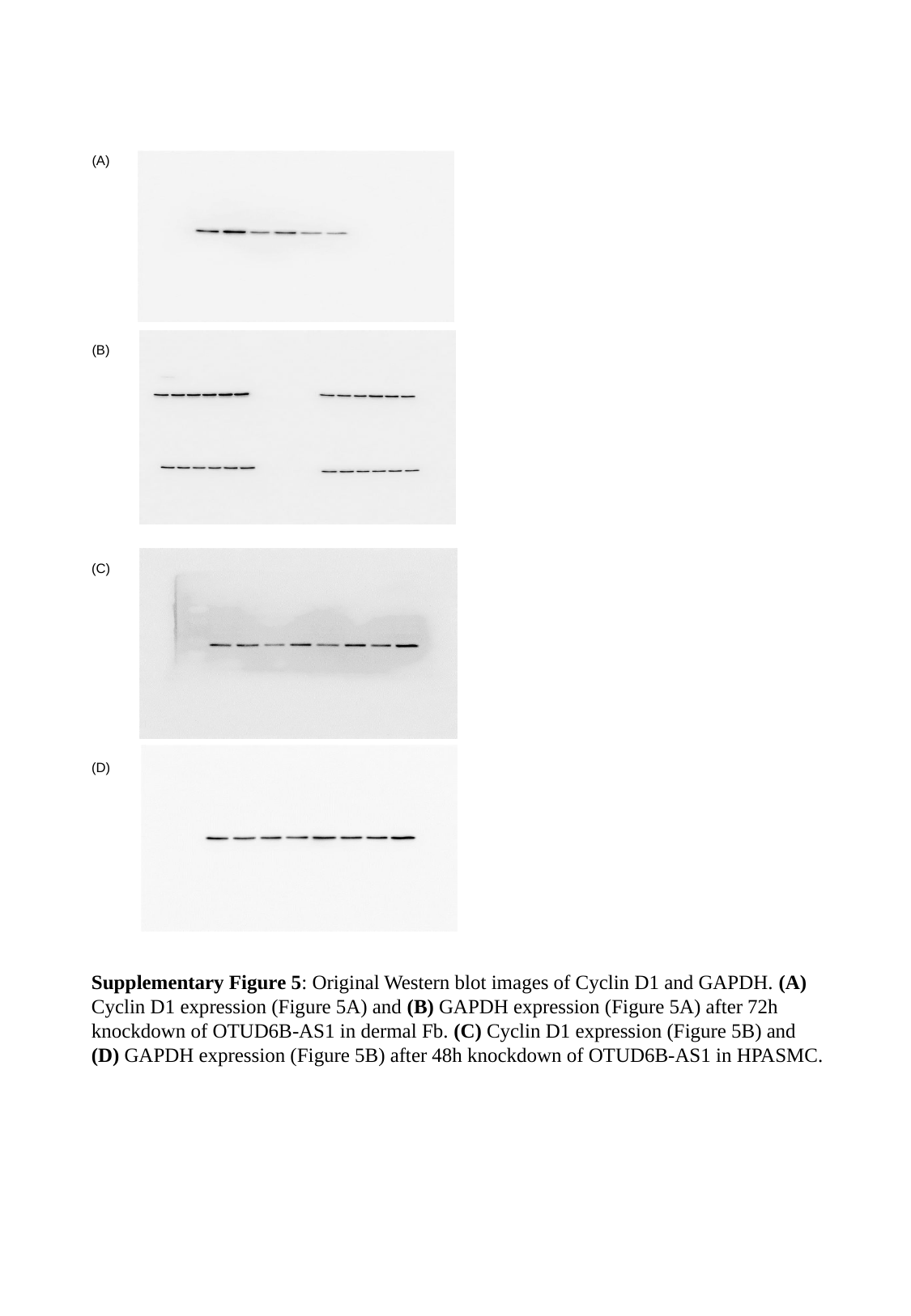

(A)
(B)
(C)
(D)
Supplementary Figure 5: Original Western blot images of Cyclin D1 and GAPDH. (A) Cyclin D1 expression (Figure 5A) and (B) GAPDH expression (Figure 5A) after 72h knockdown of OTUD6B-AS1 in dermal Fb. (C) Cyclin D1 expression (Figure 5B) and (D) GAPDH expression (Figure 5B) after 48h knockdown of OTUD6B-AS1 in HPASMC.

## Slide 6
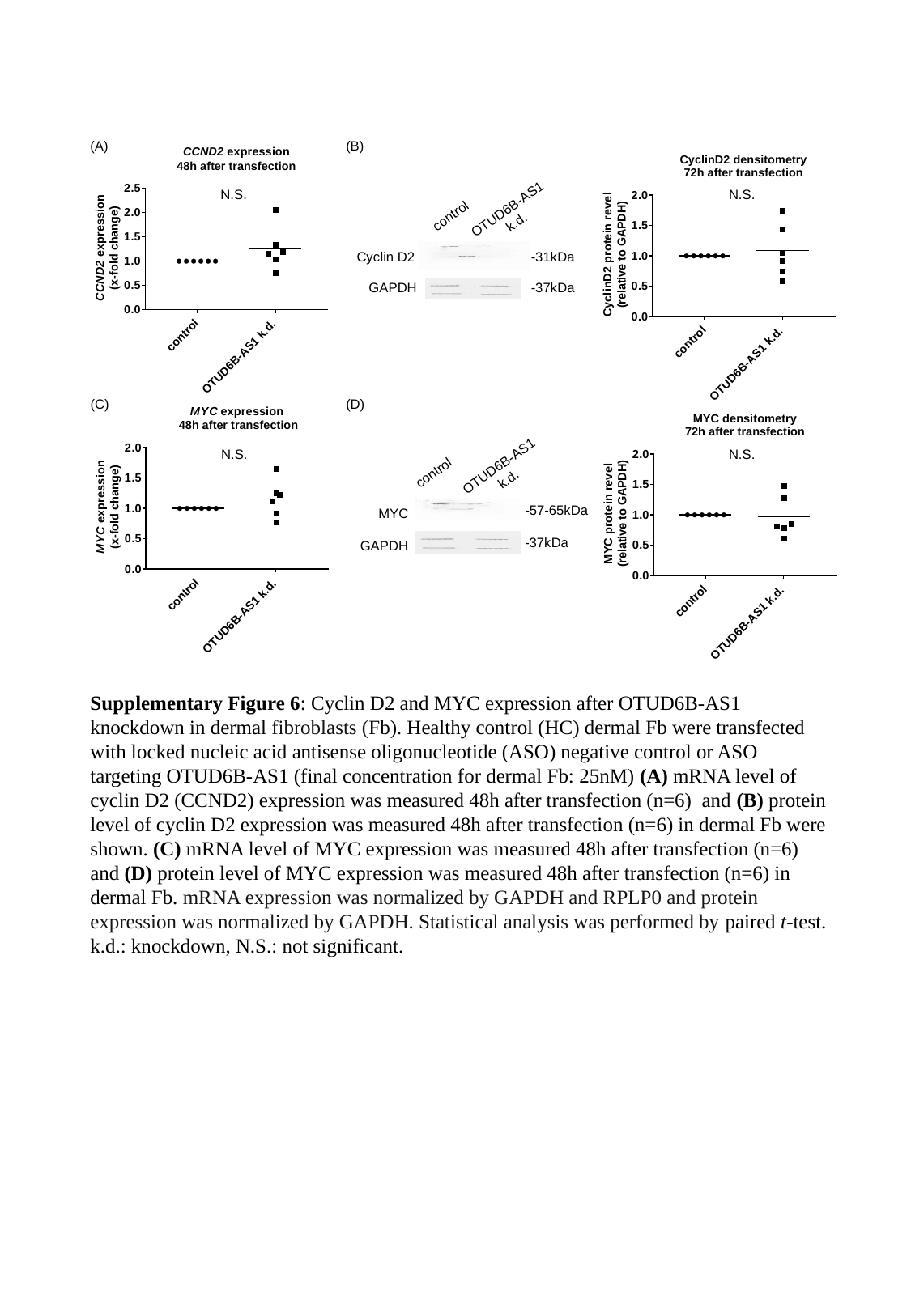

(A)
(B)
N.S.
N.S.
OTUD6B-AS1 k.d.
control
Cyclin D2
-31kDa
GAPDH
-37kDa
(C)
(D)
N.S.
N.S.
OTUD6B-AS1 k.d.
control
-57-65kDa
MYC
-37kDa
GAPDH
Supplementary Figure 6: Cyclin D2 and MYC expression after OTUD6B-AS1 knockdown in dermal fibroblasts (Fb). Healthy control (HC) dermal Fb were transfected with locked nucleic acid antisense oligonucleotide (ASO) negative control or ASO targeting OTUD6B-AS1 (final concentration for dermal Fb: 25nM) (A) mRNA level of cyclin D2 (CCND2) expression was measured 48h after transfection (n=6) and (B) protein level of cyclin D2 expression was measured 48h after transfection (n=6) in dermal Fb were shown. (C) mRNA level of MYC expression was measured 48h after transfection (n=6) and (D) protein level of MYC expression was measured 48h after transfection (n=6) in dermal Fb. mRNA expression was normalized by GAPDH and RPLP0 and protein expression was normalized by GAPDH. Statistical analysis was performed by paired t-test. k.d.: knockdown, N.S.: not significant.

## Slide 7
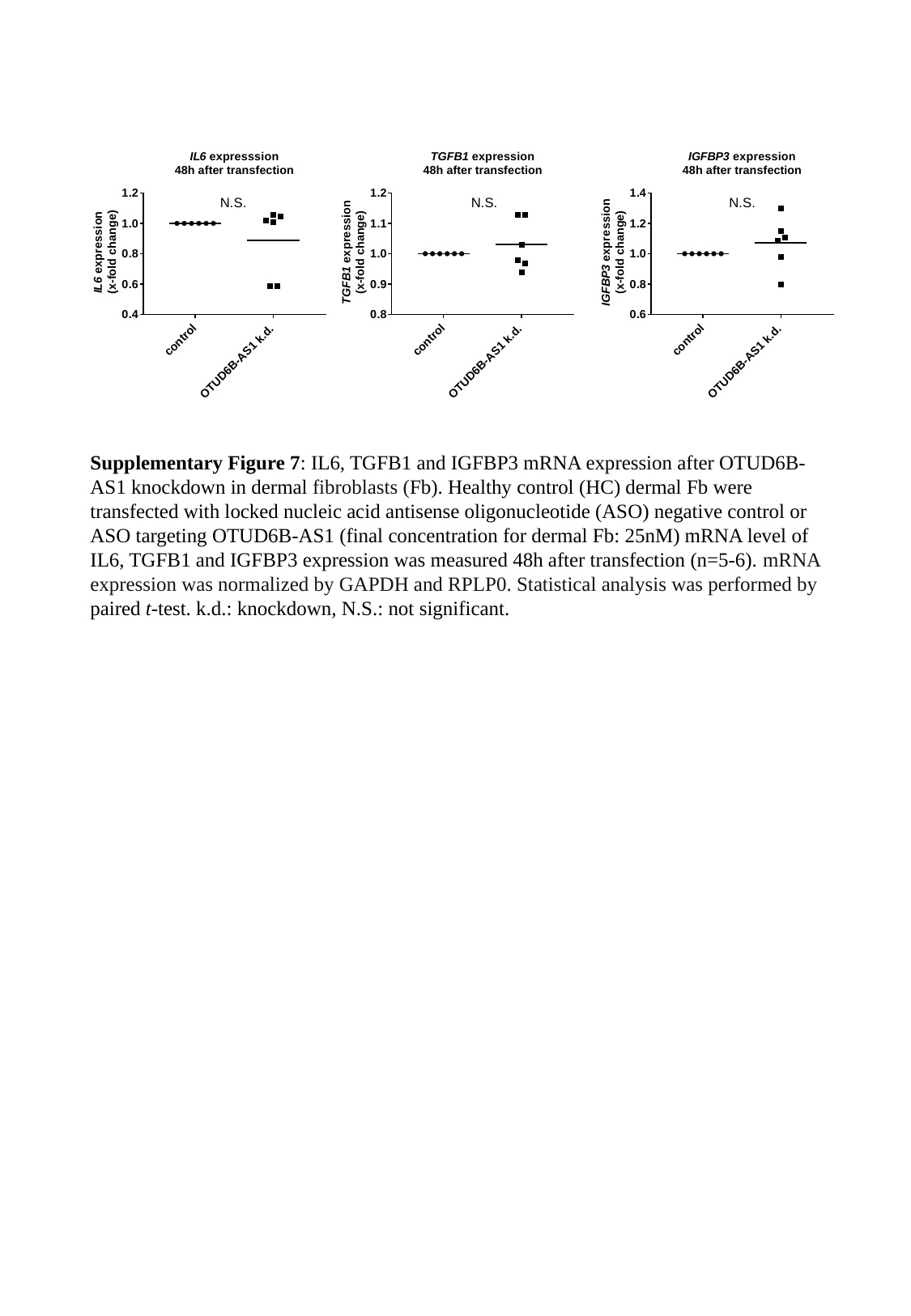

N.S.
N.S.
N.S.
Supplementary Figure 7: IL6, TGFB1 and IGFBP3 mRNA expression after OTUD6B-AS1 knockdown in dermal fibroblasts (Fb). Healthy control (HC) dermal Fb were transfected with locked nucleic acid antisense oligonucleotide (ASO) negative control or ASO targeting OTUD6B-AS1 (final concentration for dermal Fb: 25nM) mRNA level of IL6, TGFB1 and IGFBP3 expression was measured 48h after transfection (n=5-6). mRNA expression was normalized by GAPDH and RPLP0. Statistical analysis was performed by paired t-test. k.d.: knockdown, N.S.: not significant.

## Slide 8
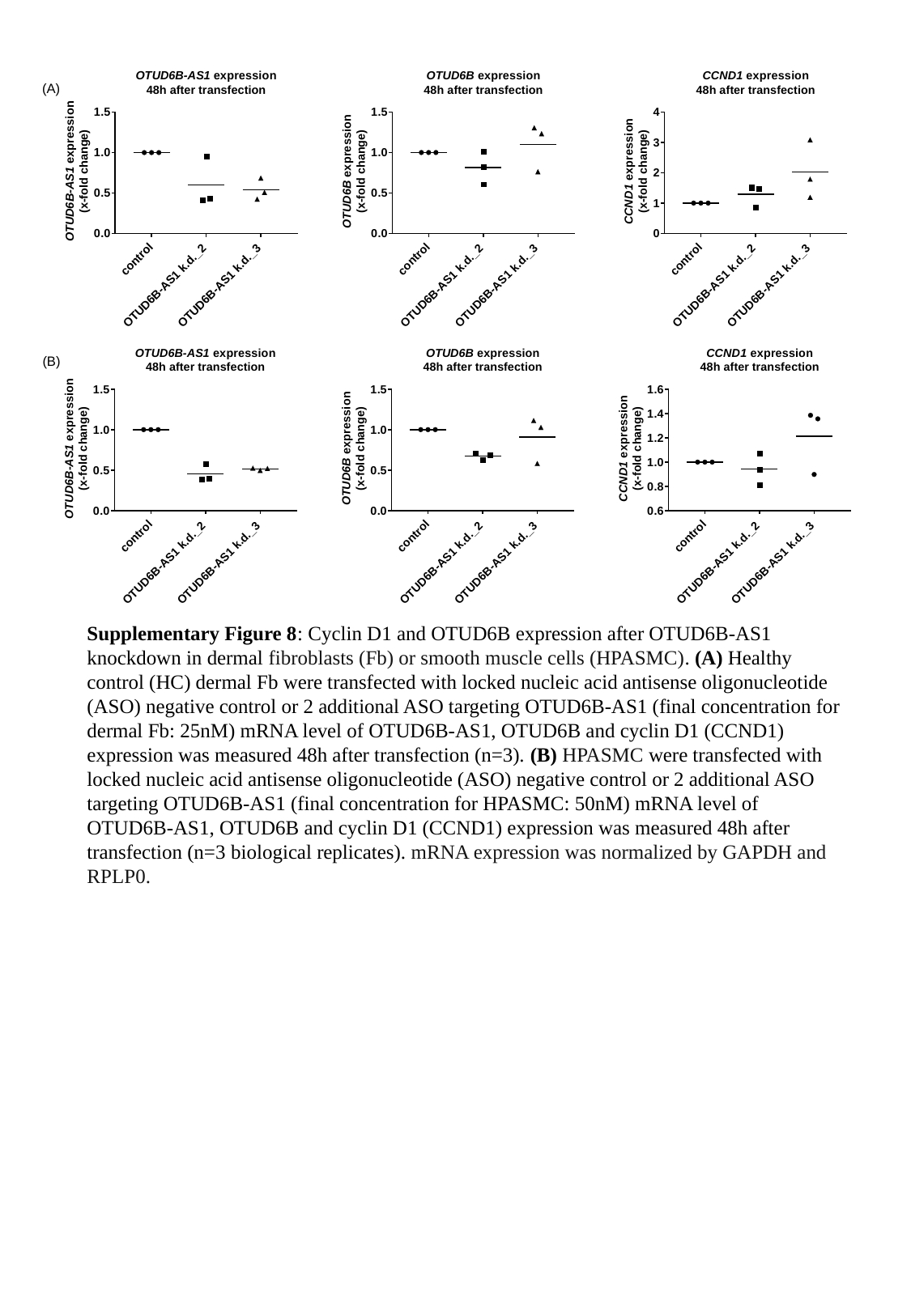

(A)
(B)
Supplementary Figure 8: Cyclin D1 and OTUD6B expression after OTUD6B-AS1 knockdown in dermal fibroblasts (Fb) or smooth muscle cells (HPASMC). (A) Healthy control (HC) dermal Fb were transfected with locked nucleic acid antisense oligonucleotide (ASO) negative control or 2 additional ASO targeting OTUD6B-AS1 (final concentration for dermal Fb: 25nM) mRNA level of OTUD6B-AS1, OTUD6B and cyclin D1 (CCND1) expression was measured 48h after transfection (n=3). (B) HPASMC were transfected with locked nucleic acid antisense oligonucleotide (ASO) negative control or 2 additional ASO targeting OTUD6B-AS1 (final concentration for HPASMC: 50nM) mRNA level of OTUD6B-AS1, OTUD6B and cyclin D1 (CCND1) expression was measured 48h after transfection (n=3 biological replicates). mRNA expression was normalized by GAPDH and RPLP0.

## Slide 9
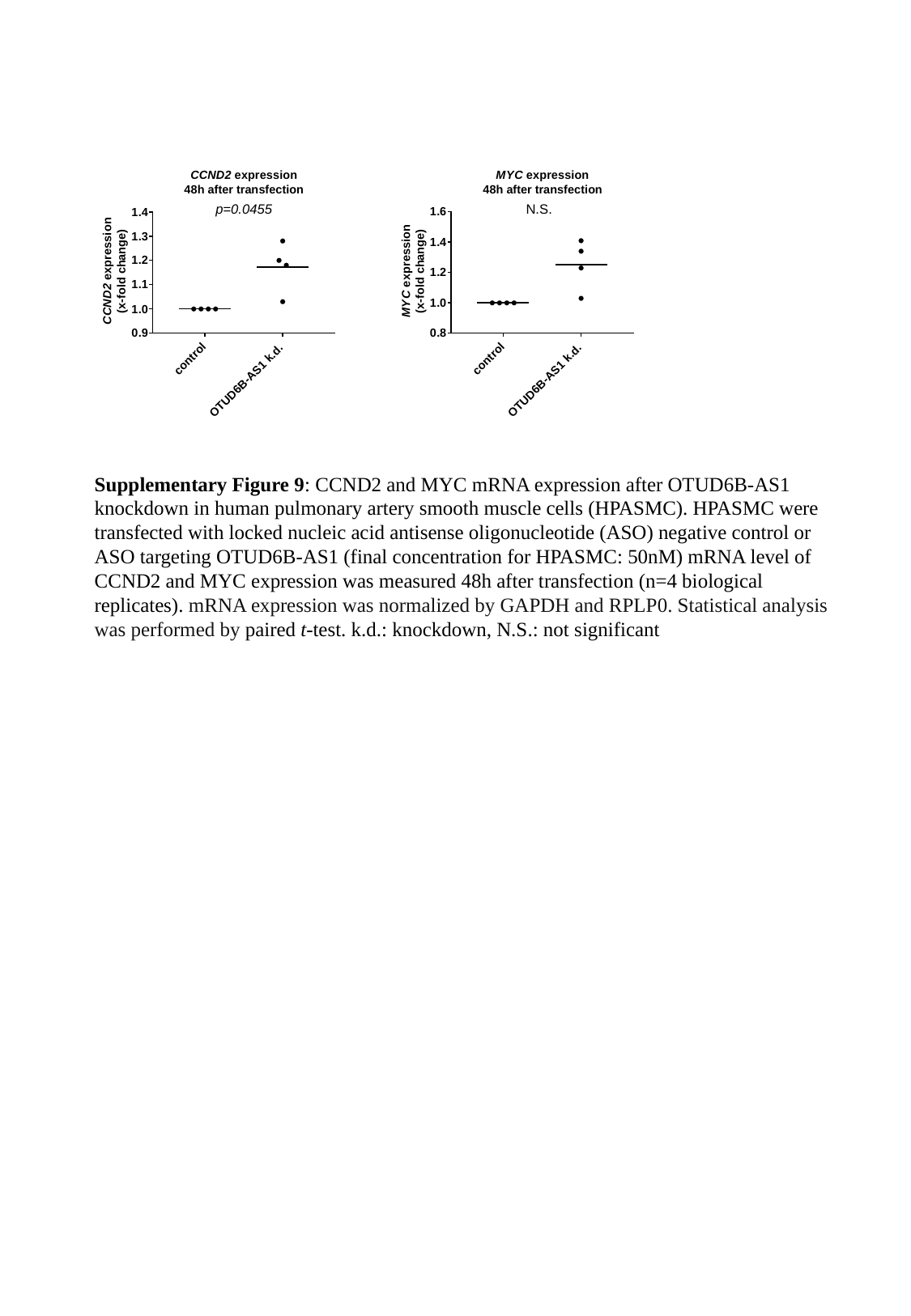

p=0.0455
N.S.
Supplementary Figure 9: CCND2 and MYC mRNA expression after OTUD6B-AS1 knockdown in human pulmonary artery smooth muscle cells (HPASMC). HPASMC were transfected with locked nucleic acid antisense oligonucleotide (ASO) negative control or ASO targeting OTUD6B-AS1 (final concentration for HPASMC: 50nM) mRNA level of CCND2 and MYC expression was measured 48h after transfection (n=4 biological replicates). mRNA expression was normalized by GAPDH and RPLP0. Statistical analysis was performed by paired t-test. k.d.: knockdown, N.S.: not significant

## Slide 10
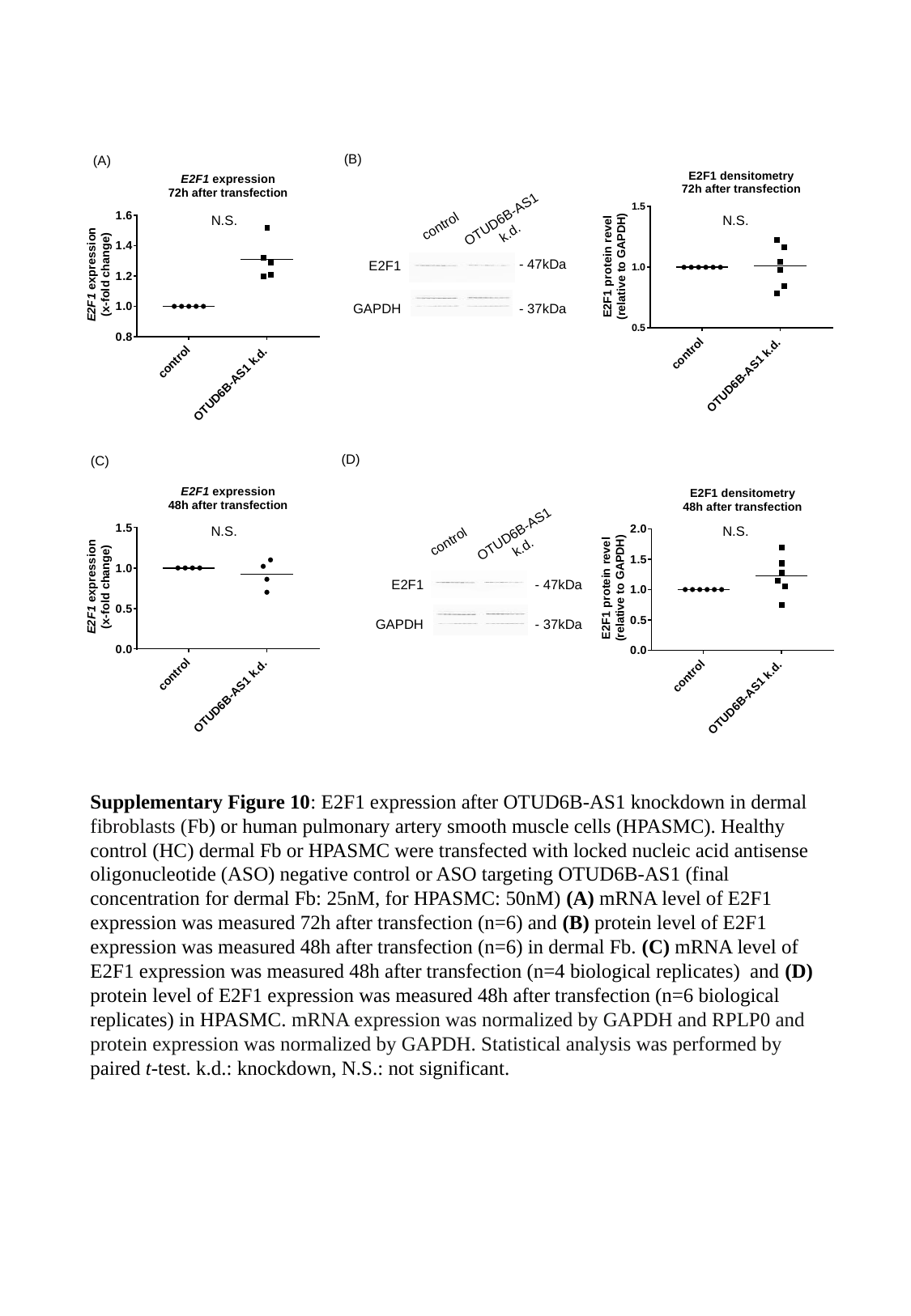

(B)
(A)
OTUD6B-AS1 k.d.
control
- 47kDa
E2F1
GAPDH
- 37kDa
N.S.
N.S.
(D)
(C)
N.S.
N.S.
OTUD6B-AS1 k.d.
control
E2F1
- 47kDa
GAPDH
- 37kDa
Supplementary Figure 10: E2F1 expression after OTUD6B-AS1 knockdown in dermal fibroblasts (Fb) or human pulmonary artery smooth muscle cells (HPASMC). Healthy control (HC) dermal Fb or HPASMC were transfected with locked nucleic acid antisense oligonucleotide (ASO) negative control or ASO targeting OTUD6B-AS1 (final concentration for dermal Fb: 25nM, for HPASMC: 50nM) (A) mRNA level of E2F1 expression was measured 72h after transfection (n=6) and (B) protein level of E2F1 expression was measured 48h after transfection (n=6) in dermal Fb. (C) mRNA level of E2F1 expression was measured 48h after transfection (n=4 biological replicates) and (D) protein level of E2F1 expression was measured 48h after transfection (n=6 biological replicates) in HPASMC. mRNA expression was normalized by GAPDH and RPLP0 and protein expression was normalized by GAPDH. Statistical analysis was performed by paired t-test. k.d.: knockdown, N.S.: not significant.

## Slide 11
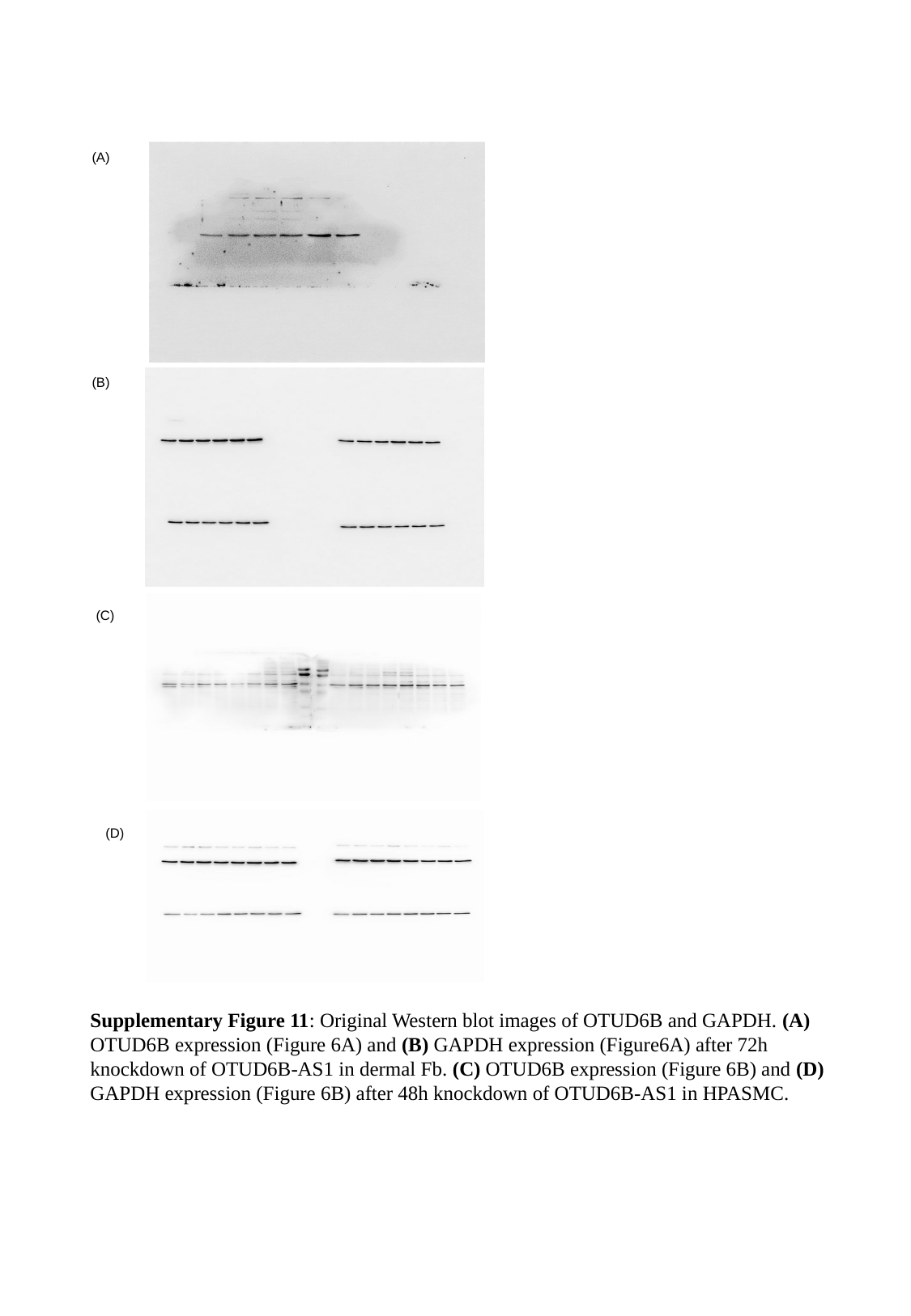

(A)
(B)
(C)
(D)
Supplementary Figure 11: Original Western blot images of OTUD6B and GAPDH. (A) OTUD6B expression (Figure 6A) and (B) GAPDH expression (Figure6A) after 72h knockdown of OTUD6B-AS1 in dermal Fb. (C) OTUD6B expression (Figure 6B) and (D) GAPDH expression (Figure 6B) after 48h knockdown of OTUD6B-AS1 in HPASMC.

## Slide 12
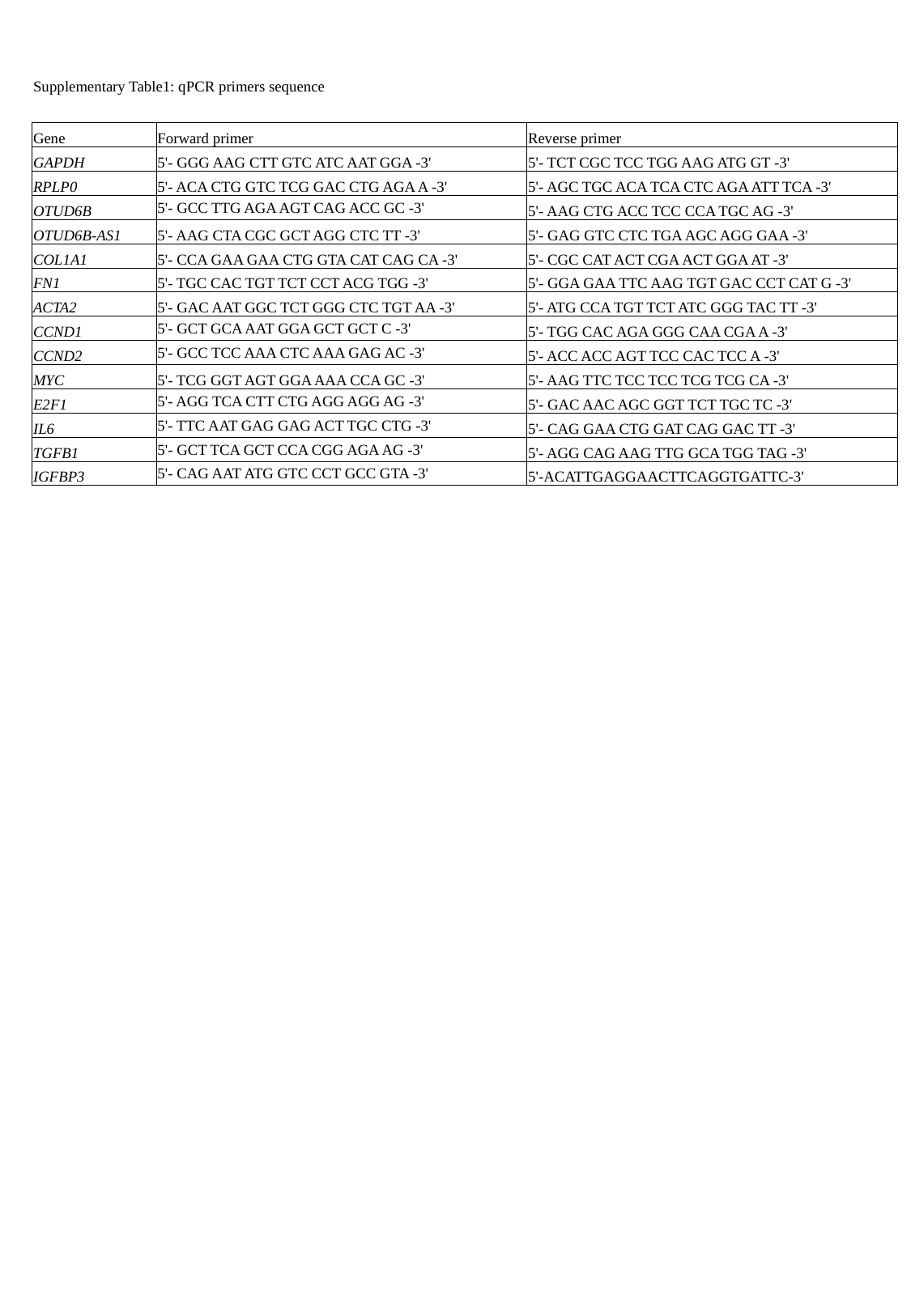

| Supplementary Table1: qPCR primers sequence | | |
| --- | --- | --- |
| | | |
| Gene | Forward primer | Reverse primer |
| GAPDH | 5'- GGG AAG CTT GTC ATC AAT GGA -3' | 5'- TCT CGC TCC TGG AAG ATG GT -3' |
| RPLP0 | 5'- ACA CTG GTC TCG GAC CTG AGA A -3' | 5'- AGC TGC ACA TCA CTC AGA ATT TCA -3' |
| OTUD6B | 5'- GCC TTG AGA AGT CAG ACC GC -3' | 5'- AAG CTG ACC TCC CCA TGC AG -3' |
| OTUD6B-AS1 | 5'- AAG CTA CGC GCT AGG CTC TT -3' | 5'- GAG GTC CTC TGA AGC AGG GAA -3' |
| COL1A1 | 5'- CCA GAA GAA CTG GTA CAT CAG CA -3' | 5'- CGC CAT ACT CGA ACT GGA AT -3' |
| FN1 | 5'- TGC CAC TGT TCT CCT ACG TGG -3' | 5'- GGA GAA TTC AAG TGT GAC CCT CAT G -3' |
| ACTA2 | 5'- GAC AAT GGC TCT GGG CTC TGT AA -3' | 5'- ATG CCA TGT TCT ATC GGG TAC TT -3' |
| CCND1 | 5'- GCT GCA AAT GGA GCT GCT C -3' | 5'- TGG CAC AGA GGG CAA CGA A -3' |
| CCND2 | 5'- GCC TCC AAA CTC AAA GAG AC -3' | 5'- ACC ACC AGT TCC CAC TCC A -3' |
| MYC | 5'- TCG GGT AGT GGA AAA CCA GC -3' | 5'- AAG TTC TCC TCC TCG TCG CA -3' |
| E2F1 | 5'- AGG TCA CTT CTG AGG AGG AG -3' | 5'- GAC AAC AGC GGT TCT TGC TC -3' |
| IL6 | 5'- TTC AAT GAG GAG ACT TGC CTG -3' | 5'- CAG GAA CTG GAT CAG GAC TT -3' |
| TGFB1 | 5'- GCT TCA GCT CCA CGG AGA AG -3' | 5'- AGG CAG AAG TTG GCA TGG TAG -3' |
| IGFBP3 | 5'- CAG AAT ATG GTC CCT GCC GTA -3' | 5'-ACATTGAGGAACTTCAGGTGATTC-3' |
